# Cation Adsorption in TiO_2_ Nanotubes: Implication for Water Decontamination

**DOI:** 10.1021/acsanm.3c00916

**Published:** 2023-07-11

**Authors:** Atiđa Selmani, Bertrand Siboulet, Mario Špadina, Yann Foucaud, Goran Dražić, Borna Radatović, Karla Korade, Ivan Nemet, Davor Kovačević, Jean-François Dufrêche, Klemen Bohinc

**Affiliations:** † Division of Physical Chemistry, Ruđer Bošković Institute, Bijenička Cesta 54, 10000 Zagreb, Croatia; ‡ Pharmaceutical Technology & Biopharmacy, Institute of Pharmaceutical Sciences, University of Graz, A-8010, Graz, Austria; ¶ ICSM, Université Montpellier, CEA, CNRS, ENSCM, 30207 Bagnols-sur-Ceze, France; § Faculty of Health Sciences, University of Ljubljana, Zdravstvena 5, SI-1000 Ljubljana, Slovenia; ∥ Laboratory for Materials Chemistry, National Institute of Chemistry, Hajdrihova ulica 19, SI-1000 Ljubljana, Slovenia; ⊥ Institute of Physics, Bijenicka 46, 10 000 Zagreb, Croatia; # Faculty of Science, University of Zagreb, Horvatovac 102A, 10 000 Zagreb, Croatia

**Keywords:** adsorption, charge inhomogeneities, nanotubes, potentiometric acid−base titration, surface charge

## Abstract

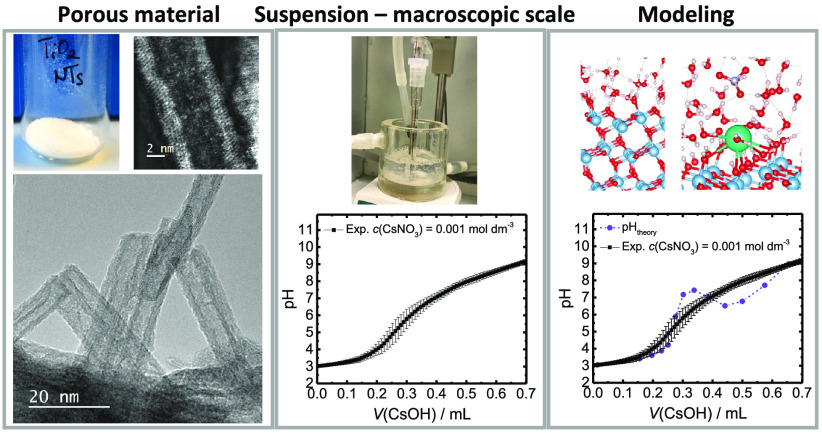

TiO_2_ nanotubes constitute very promising nanomaterials for water decontamination by the removal of cations. We combined a range of experimental techniques from structural analyses to measurements of the properties of aqueous suspensions of nanotubes, with (i) continuous solvent modeling and (ii) quantum DFT-based simulations to assess the adsorption of Cs^+^ on TiO_2_ nanotubes and to predict the separation of metal ions. The methodology is set to be operable under realistic conditions, which, in this case, include the presence of CO_2_ that needs to be treated as a substantial contaminant, both in experiments and in models. The mesoscopic model, based on the Poisson–Boltzmann equation and surface adsorption equilibrium, predicts that H^+^ ions are the charge-determining species, while Cs^+^ ions are in the diffuse layer of the outer surface with a significant contribution only at high concentrations and high pH. The effect of the size of nanotubes in terms of the polydispersity and the distribution of the inner and outer radii is shown to be a third-order effect that is very small when the nanotube layer is not very thick (ranging from 1 to 2 nm). Besides, DFT-based molecular dynamics simulations demonstrate that, for protonation, the one-site and successive association assumption is correct, while, for Cs^+^ adsorption, the size of the cation is important and the adsorption sites should be carefully defined.

## 1 Introduction

Human activities in industry, technology, and energy production generate various contaminants, such as organic compounds, dyes, radionuclides, or heavy metals, which entail a serious hazardous threat to life in general. Those emerging pollutants are difficult to remove, and therefore, finding new alternatives for water remediation is highly required. As instance, the presence of radionuclides in the aquatic ecosystem can lead to serious hazardous events in the environment due to their long half-life. The nuclear waste management process usually employs chemical methods, which involve chelator ligands that bind metal cations and remove them from wastewater. However, the undesired consequence is the production of waste, which has to be further processed. The new strategies for water remediation are based on carbon,^1^ polymeric^2^ and organic materials,^3^ inorganic composites,^4^ and hybrid inorganic–organic materials.^5−7^ The aforementioned materials are employed as adsorbents, photocatalysts, and biocatalysts in water recovery processes. Although numerous methods are available for water remediation, adsorption has been recognized as a method of choice, due to its efficiency, simple design, and performance. The adsorption on porous nanomaterials (NMs) serves as a promising strategy for the selective removal of cations. In the context of water decontamination by selective removal of cations, especially in the framework of the nuclear industry, cesium^8^ and strontium^9^ are important target metal ions for which porous nanomaterials have a considerable potential.^10^ Among all the existing metal–oxide nanotubes, very interesting are the titanium oxide nanotubes (TiO_2_ NTs) due to their nontoxicity, low-cost synthesis, high photocatalytic activity, and chemical stability, as well as their reusability for multiple decontaminations and stripping cycles.^11^ Moreover, they present very good adsorption properties due to large specific surface areas and to the fact that both inner and outer surfaces are in contact with the interacting medium. Their amphoteric character enables tuning the solute adsorption with respect to ambient pH and composition of the colloidal suspension of nanotubes.^12^ For all these reasons, TiO_2_ NTs are a very promising improvement from existing composite materials.

The fundamental knowledge of the adsorption properties of nanoporous nanomaterials is crucial for optimizing their applications such as metal decontamination,^13^ photocatalysis,^14^ organic molecules removal or decomposition,^15^ etc. Numerous studies investigated the adsorption and decontamination efficiencies of loose nanotube materials.^13^ From a fundamental point of view, it is still unclear whether the solute adsorption is equivalent between the inner and outer nanotube surfaces exposed to the liquid medium. Furthermore, it is not evident if solutes are chemi- or physisorbed on the nanotube surfaces at thermodynamic equilibrium. However, it is of paramount interest to understand these two questions before designing any practical process at the laboratory scale or at the pilot scale. To access the equilibrium properties, the classical approaches usually employ Langmuir-like models, in which all the surfaces are treated equally.^13,16^ While this is a very good approximation for the gas adsorption by nanotubes or by other porous materials,^17^ it is not sufficient when the objective is to assess the preferential adsorption of solutes on the nanotubular structures in a liquid medium. Moreover, in the case of a higher affinity for adsorption of solutes on either inner or outer nanotubes surface, it would impact process efficiencies beyond adsorption/decontamination, but including doping, photocatalysis, and contaminants degradation, *etc*. This is why further studies in this domain are required.

In the context of studying the efficiency of adsorption by nanomaterials, batch adsorption experiments are used in most cases. Nevertheless, the resulting adsorption efficiency from the batch experiment in the solid state should not be confused with the properties of the aqueous suspension, where the nanomaterial is in equilibrium with all ions and water. Apart from directly at the nanomaterial surface, ions reside in the electric double layer (EDL).^18^ To assess the adsorption properties in the aqueous suspension, the activity of protons, also called the proton consumption, is measured during the titration experiments and can be directly compared to the mesoscopic models that take into account the local equilibria between the solution and the exposed surfaces of the adsorbent.^19,20^ For the amphoteric nanomaterials, it was also shown that the size and curvature of the EDL are important. While a lot of work has been done on addressing the charge properties for variable sizes of TiO_2_ or SiO_2_ nanoparticles,^21,22^ considerations are limited to the average size in terms of lengths and radius for nanotubular structures. Moreover, in the case of TiO_2_ NTs, the distributions of inner and outer radii and lengths can be severely polydisperse.^23^ Overall, the usual route to probe the adsorption of solutes from the aqueous suspension is to combine potentiometric acid–base titration experiments with surface complexation (SC) models.^24^ SC models are usually merged with classical density functional theory (cDFT), or simple Poisson–Boltzmann.^25^ The idea is always the same: to connect analytical chemistry speciation by multiple equilibria (SC models) with the spatial distribution of ionic species in EDL in the presence of the nanomaterial. The general framework is very powerful since it has been applied consistently (with upgrades) for decades now, with applications in colloidal chemistry, geochemistry, nanopores, biophysics, and many other applied chemistry fields.^26,27^

While the technologies that use nanoporous materials are yet to ripen, pilot and larger scales processes will operate in air atmosphere conditions.^28^ This means that CO_2_ is always present in separation processes and can therefore be considered as an impurity or as a contaminant.^20^ Nevertheless, the effect of CO_2_ is seldom included or rationalized in most models. In the case of the TiO_2_ NTs, the study based on the SC-cDFT approach reported preferentially higher proton adsorption on the inner nanotubes surfaces at equilibrium, which led to a higher charge on the outer surfaces.^29^ The predicted charging mechanism demonstrated a strong influence of the bulk pH and salt concentration. While, for these amphoteric materials, the adsorption is primarily governed by the electrostatic interactions between solute and adsorbent, the mesoscopic effects of the solutes confinement as well as the curvature of nanotubes also presented a significant effect.^23^ Besides mean-field mesoscopic models, molecular dynamics simulations can bring useful insights into the adsorption mechanisms at a molecular level,^30−32^ which are of uttermost importance to characterize the Cs^+^ adsorption on TiO_2_ NTs. Considering the significant reactivity of TiO_2_ surfaces in the presence of water, particularly in terms of acid–base reactions, density functional theory (DFT) based molecular dynamics (DFT-MD) simulations represent a very efficient compromise between the accuracy, including the description of the reactivity, and the computational costs.^33,34^

In this study, by a systemic experimental and theoretical work, we encompass different time and length scales to describe the charge inhomogeneities of TiO_2_ NTs in aqueous suspensions in terms of all relevant constituents and resulting chemical equilibria (ions and nanotubes). We used high-resolution transmission electron microscopy (HR-TEM) and atomic force microscopy (AFM) to assess the structural properties of TiO_2_ NTs and electrophoretic mobility measurements, potentiometric acid–base titrations, and batch adsorption experiments to probe the cation adsorption of TiO_2_ NTs in aqueous suspensions. The experiments are combined with two different scales of modeling to address the question of the preferential adsorption of Cs^+^ and similar solutes on the exposed nanotube surfaces: a mesoscopic Poisson–Boltzmann electrostatic description and DFT-based molecular dynamics simulations. The objective is to derive a feasible model and to compare it with macroscopic experiments such as potentiometric acid–base titrations, in which the total amount of ions is changed with the addition of the titrant. Meanwhile, we include the effects of dissolved CO_2_ that can act as a contaminant and can be a significant contributor to charge properties in both models and the experiments.

## 2 Experimental Section

### 2.1 Materials

**Figure 1:**
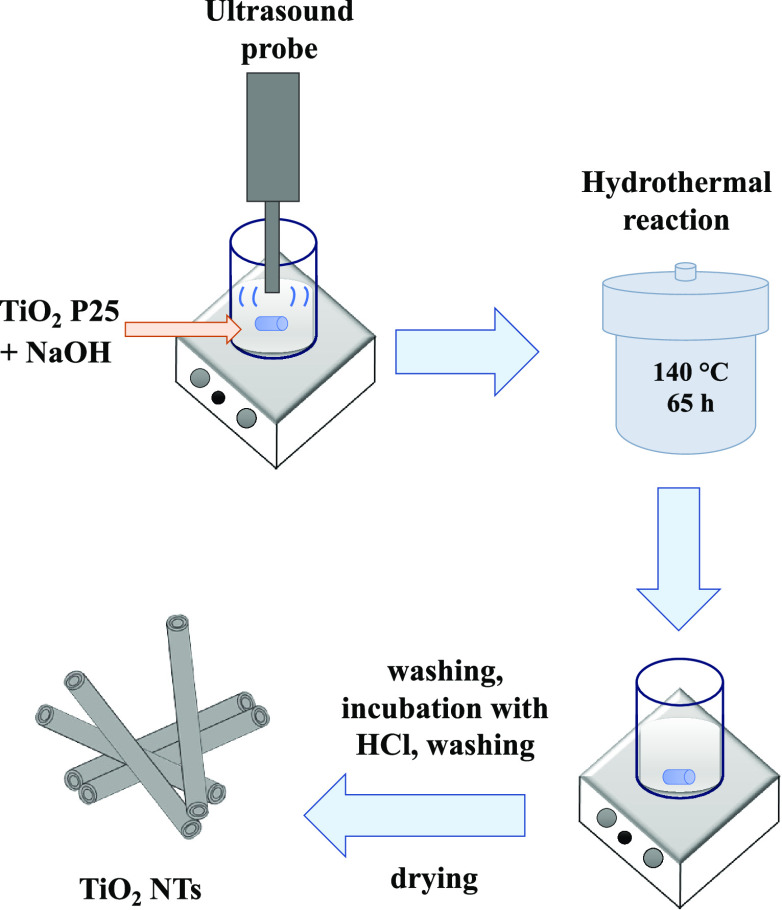
Synthesis of TiO_2_ nanotubes.

The synthesis of TiO_2_ NTs was based on TiO_2_ P25 (75% Anatase, 25% Rutile Degussa) and is depicted in Figure 1. The nitric acid (HNO_3_), sodium hydroxide (NaOH) and cesium hydroxide (CsOH) were provided by Riedel de Haën. Cesium nitrate (CsNO_3_) was purchased from Fluka. Potassium hydrogen phthalate (KHP) used for the NaOH standardization was obtained from Sigma-Aldrich. Five standard buffers, pH = 2, 4, 6, 8, and 10 that were purchased from Reidel-de Haën were used for the potentiometric measurements, i.e., electrode calibration. All solutions were prepared by dissolving them in CO_2_-free deionized water. All chemicals were used as-received without purification.

### 2.2 Methods

TiO_2_ NTs were fabricated as reported in our previous studies.^23^ The composition and structural properties of TiO_2_ NTs were determined with powder X-ray diffraction (PXRD) and attenuated total reflectance Fourier transform infrared spectroscopy (ATR FTIR). The morphological features were obtained via HR-TEM, SEM, and AFM. The specific surface area was determined with a multipoint Brunauer–Emmett–Teller method. The acid–base surface properties were elucidated by means of acid–base potentiometric titrations and electrokinetic measurements, for which all chemicals were used as-received without purification. To test the adsorption affinity of cesium ions for the TiO_2_ NTs surface, we performed adsorption measurements.

Due to the number of methods performed for this study, a detailed description of the experimental part is given in the Supporting Information.

## 3 Theory and Atomistic Simulations

### 3.1 The Mesoscopic Model for Theoretical Description of TiO_2_ NTs Charge Properties and Cation Adsorption

We propose a mesoscopic method to simulate the behavior of ions around solid amphoteric nanotubes, in this case, TiO_2_ NTs, during the potentiometric acid–base titration experiment under an air atmosphere. The method is exhaustive, in the sense that all experimental quantities are simulated in a self-consistent way. These quantities are the pH, the total quantities of all ions and the surface sites of amphoteric nanotubes. Our method allows adjusting a small number of unknown parameters of the system, those which characterize the adsorption properties of the ions. An output of the results is also the determination of the ion spatial distributions between all locations of the system: inside the tubes, on the surfaces, and outside the tubes. We also handle CO_2_ “contamination”, inserting reference titration results, namely, TiO_2_ free titrations, into the titration simulations. To our knowledge, such a model has never been published.

To represent the CsNO_3_ aqueous suspension of TiO_2_ NTs, we consider an infinitely long tube immersed in an ideal gas of ions with dielectric properties of the medium being expressed by the dielectric constant ϵ_r,1_. The experimental conditions of potentiometric acid–base titrations were (a) low suspension mass density (γ = 1 g dm^–3^) and (b) moderate CsNO_3_ concentrations (*c*(CsNO_3_) from 0.001 mol dm^–3^ to 0.01 mol dm^–3^). To simplify the mode, we neglected the interactions between TiO_2_ NTs based on the argument that these latter are sufficiently diluted, and electrostatic potentials for moderate salt concentrations are screened over a shorter distance compared with the average distance between NTs.

**Figure 2:**
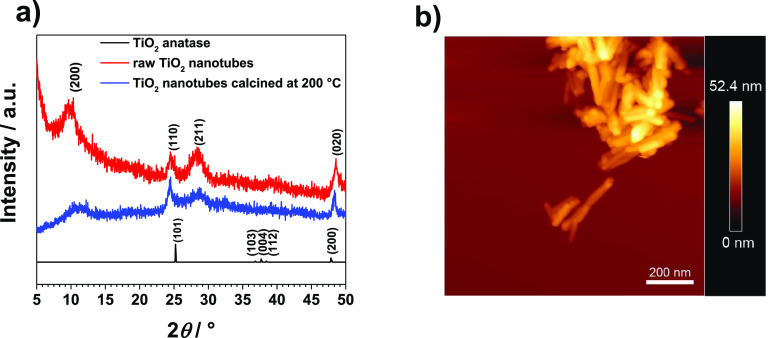
Characterization of nanotubes sample. (a) Powder X-ray diffraction patterns of the TiO_2_ powders used in this study. (b) Topography (height) image of annealed TiO_2_ NTs sample.

To represent the amphoteric character of TiO_2_ NTs, the model NTs surface charge was modulated via a charge regulation mechanism.^35^Figure S8 (see Supporting Information) shows the schematic representation of the charging processes that take place at both TiO_2_ NTs surfaces. Both surfaces have the same site density and the same type of sites and are in contact with the aqueous solution containing ions. The dielectric properties of the aqueous solution, ϵ_r,1_, and of the TiO_2_ solid, ϵ_r,2_, are considered as equal (ϵ_r,1_ = ϵ_r,2_ = 80.1). We considered anatase planes based on structural data (see Figure 2). By considering only anatase, we simplify the model. The heterogeneity of the TiO_2_ NTs samples^36^ in terms of the presence of amorphous solid, different exposed atoms, the polydispersity of sizes, *etc*. mandates simplification of the model, which reduces the number of adjusted parameters.^37^ We focused on the preferential cation adsorption between the exposed surfaces, for which we considered a single adsorption site along with multiple equilibria model for successive protonations and Cs^+^ associations onto the exposed oxygen atom.^38^ We obtained the following set of equilibria, expressed as site-ion association constants:
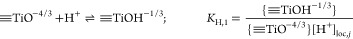
1
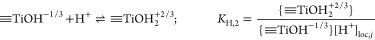
2
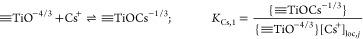
3
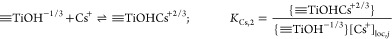
4where [H^+^]_loc,*j*_ and [Cs^+^]_loc,*j*_ are local (surface) ions concentrations. Index *j* depicts either the inner or outer nanotube surface. Local concentrations are functions of the electrostatic potential at the surfaces ψ(*r* = *R*_inner_) or ψ(*r* = *R*_outer_),^39^*K*_H,1_ and *K*_H,2_ are successive surface protonation reactions, and *K*_Cs,1_ and *K*_Cs,2_ correspond to Cs^+^ association constants. The interactions of the surface groups are taken into account indirectly through the mean field approximation.^40^ The surface sites density Γ is defined as
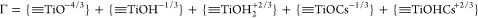
5It follows that the surface charge densities are defined as σ_*j*_ = *e*Γ∑_*x*=1_^5^*z*_*x*_*f*_*x*,*j*_., where *e* is the elementary charge, *z*_*x*_ represents charges of sites *x* (eq 5), and *f*_*x*,*j*_ are populations of the site *x* on the surface *j*. *f*_*x*,*j*_ values are obtained by combining eq 1 to 4. Full expressions for *f*_*x*,*j*_ are given in the Supporting Information.

Besides, to represent the effect of the CO_2_ as a contaminant in acid–based titrations of nanomaterials suspension, we considered the following set of chemical equilibria:
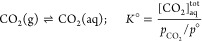
6
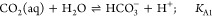
7
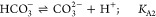
8where *K*_A1_ and *K*_A2_ are carbonates successive dissociation constants. The objective of the model is to calculate the total concentration of dissolved CO_2_ in the solution or suspension , as a function of pH. Once the quantity  as a function of pH is known, HCO_3_^–^ and CO_3_^2–^ speciation can be imported in the total proton balance calculation of the system. By considering this, we considered the contaminant at any point within the potentiometric titration experiments. This is the route toward applying mesoscopic models for real application systems of TiO_2_ NTs (and metal oxides in general) under an air atmosphere that have the potential for scaling to the pilot process.  is fitted from the blank titration and it is assumed that, for short time intervals, the dissolution rate of CO_2_ in the aqueous medium is the same for CsNO_3_ aqueous solution and for the dilute suspension containing TiO_2_ NTs. The full model derivation and the fitting of the blank titration procedure are given in detail in the Supporting Information.

To account for the electrostatic interactions in the system composed of a single, infinitely long TiO_2_ NT immersed in the aqueous solution containing the ions, we adopted the classical Density Functional Theory (c-DFT) of inhomogeneous Coulomb fluids.^41^ We considered a cell model in which the infinitely long TiO_2_ NT is placed.^41,42^ Ions were considered as an ideal gas under the influence of an external electric field arising from the TiO_2_ NT surface. Ions interacted with both the inner and outer surface of the nanotube and ion–ion interactions were Coulombic only. The minimization of the grand canonical potential functional yielded one-body ion densities ρ_α_(*r*) = ρ_α_^0^ exp(−*z*_α_Φ(*r*)), where ρ_α_^0^ is the concentration of the ion α in the bulk (the reservoir), *z*_α_ is the charge of that ion, and Φ(*r*) = *e*Ψ(*r*)/*k*_B_*T* is the “dimensionless” potential. Ψ(*r*) is the electrostatic potential, *e* is the elementary charge, *k*_B_ is the Boltzmann constant, and *T* is the thermodynamic temperature.^43^ By introducing ion densities into the Poisson equation and considering only the radial component of the Laplace operator in the cylindrical coordinate system, one can derive the Poisson–Boltzmann level of description for the infinitely long nanotube:
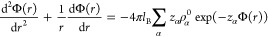
9where *l*_B_ = *e*^2^/4*πk*_B_*Tϵ*_0_ϵ_r,1_ is the Bjerrum length and ϵ_0_ is the vacuum permittivity. Noteworthily,  is the inverse Debye length. We considered that  was 0 at *r* = 0 due to the symmetry (zero charge in the middle of the nanotube) and that the total charge was 0 at *r* = +*∞*. Using the continuity equation, we obtained the electrostatic potential through the TiO_2_ NTs layer in the following expression:
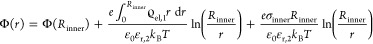
10The full model derivation is given in the Supporting Information.

To summarize, the model presented in this work is an extension of the theoretical framework first derived to qualitatively explain the results of colloidal polyelectrolyte titration of TiO_2_ NTs.^29^ The three key differences here are (i) the inclusion of Cs^+^ association and competition reactions, (ii) the consideration of dissolved HCO_3_^–^ and CO_3_^2–^ species, and (iii) the calculations that were made in the semigrand canonical ensemble, which means that we can reproduce the potentiometric acid–base experiments directly (only water molecules are considered as intensive quantity by high dilution).

### 3.2 Numerical Implementation of the Mesoscopic Model

We simulate the global adsorption system numerically. The simulation intends to mimic closely the experimental process: CsOH is added steadily to the initial CsNO_3_ aqueous suspension of TiO_2_ NTs. While the pH is measured for any addition of a volume of the base, *V*_d_(CsOH), Cs^+^, NO_3_^–^, H^+^, and OH^–^ concentrations are known from the experiment and simulated from the code. The simulation includes self-consistent potential and concentration profiles. Concentrations include Cs^+^, NO_3_^–^, H^+^, and OH^–^ species, both in the aqueous medium and on the inner and the outer NTs surfaces. We proceed to iterations at two levels: (i) on the electric potential (inner loop) and (ii) on the concentrations (outer loop). Each guess for Cs^+^ and NO_3_^–^ concentrations at a long distance from the nanotube generates a simulated pH through electroneutrality. The concentration of ions anywhere in space is the product of the bulk concentration and the Boltzmann factor. We proceeded to the inner iteration loop. Each iteration loop starts with a guess of the potential at the center of the tube and ends with the electric potential over a long distance (the bulk value). Inner iterations stop when the potential is negligibly small over a long distance. The total Cs^+^ and NO_3_^–^ are then calculated and sent to the outer loop. The following iterations proceed with new guesses about Cs^+^ and NO_3_^–^. We resume outer loop iteration until the total concentrations match the experimental values. We underline that using electroneutrality for the determination of the pH is critical at neutral pH since the concentrations of both H^+^ and OH^–^ ions can be small. Note that the total quantity of ions is the sum of the concentrations at a long distance, adsorbed ions at both surfaces, and in double layers both inside and outside of nanotubes. This is how we fulfill the criteria for the spontaneous adsorption of Cs^+^ and H^+^ through iterations. The details of the numerical implementation are given in detail in the Supporting Information.

### 3.3 Atomistic Simulations

All the calculations were conducted using the Vienna Ab Initio Simulation Package.^44^ The semilocal exchange-correlation PBE functional in the generalized gradient approximation of Perdew and co-workers was used^45^ along with the D2 method of Grimme to take into account the dispersion forces.^46^ The electron–ion interactions were described using the projector augmented wave method^47,48^ with a plane-wave cutoff kinetic energy of 400 eV. The following valence electrons were considered: 1s^1^ for H, 2s^2^2p^4^ for O, 3s^2^3p^6^4s^1^3d^3^ for Ti, 2s^2^2p^3^ for N, and 5s^2^5p^6^6s^1^ for Cs. All the calculations were performed using the Γ-point only due to the large size of the cell and the significant disorder of atoms. The Kohn–Sham equations^49,50^ were solved self-consistently^51^ until the difference in energy between cycles was lower than 10^–4^ eV. For the relaxation of the first primitive cell and of the slab prior to the DFT-MD simulations, the calculations were conducted using a 4 × 4 × 4 *k*-points grid with a kinetic cutoff energy of 1,000 eV, an energy threshold of 10^–8^ eV, and the relaxation was stopped when all forces were lower than 10^–3^ eV· A^–1^. Since the semilocal DFT exchange-correlation functionals such as PBE are known to inaccurately describe the high-correlated nature of Ti 3d electrons, we used the DFT+U formalism of Dudarev,^52^ as implemented in VASP. The on-site Coulomb repulsion of the Ti 3d electrons was described using *U* = 3.50 eV (and *J* = 0.00 eV), considering most values used in the literature.^53−56^ The *NVT* DFT-MD simulations were conducted using a Nosé–Hoover thermostat^57−59^ at a temperature of 300 K with a time step of 1 fs, allowed by replacing the mass of the proton by that of the tritium isotope. Each DFT-MD simulation was conducted during 80 ps, and a thermalization period of 10 ps was excluded at the beginning of the simulation.

A primitive cell of anatase was generated following the experimental cell parameters *a* = *b* = 3.7842 Å, *c* = 9.5146 Å, and α = β = γ = 90° given by Horn and co-workers.^60^ This quadratic cell was relaxed in terms of ion positions, cell shape, and cell size, and the fully relaxed primitive cell exhibited *a* = *b* = 3.9344 Å, *c* = 9.7244 Å, and α = β = γ = 90°, which was in good accordance with the experimental parameters. Based on this relaxed primitive cell, we generated a supercell and created the (101) surface, which is known to be the most exposed surface of anatase.^53,61−63^ The obtained slab was composed of three layers of 12 titanium atoms, which therefore represented a total number of 36 titanium atoms and 72 oxygen atoms (Figure S15a). On the (101) surface, among the 12 titanium atoms that constituted the surface plane, six were five-coordinated and located slightly above the average surface plane, while the six others were six-coordinated, like in the TiO_2_ bulk, and located slightly below the average surface plane. Besides, the in-plane surface oxygen atoms were three-coordinated, like in the TiO_2_ bulk, while six oxygen atoms were significantly above the average plane and were only two-coordinated. The ions positions of this slab was relaxed by a static DFT calculation that consisted in a series of wave function optimizations. To avoid any unwanted interaction between the two reciprocal surfaces (whose existence is due to the periodic boundary conditions), a vacuum of 20 Å was added above the uppermost atom of the TiO_2_ slab along the *z*-axis. Then, explicit water molecules were randomly added using the packmol software,^64^ to completely fill in the aforementioned vacuum by meeting a density of 1 g cm^–3^ for the added liquid phase. The DFT-based molecular dynamics simulations were conducted on this system. For the simulations performed with CsNO_3_, the latter was added close to the surface by substituting four water molecules. All the molecular structures were visualized using VESTA software.^65^

## 4 Results

### 4.1 Synthesis and Characterization

An alkaline hydrothermal synthesis route was applied to produce TiO_2_ NTs. TiO_2_ anatase in 10 mol dm^–3^ NaOH was treated for 65 h at 140 °C. The annealing post-treatment of the TiO_2_ NTs was performed in order to investigate the effect of the annealing temperature on the crystallinity of TiO_2_ NTs. PXRD was employed to determine the composition and crystal structure of the TiO_2_ NTs. The obtained PXRD diffraction pattern of the TiO_2_ NTs corresponds to protonated titanate, H_2_Ti_3_O_7_ (Figure 2a). Besides, the specific surface areas for raw and annealed TiO_2_ NTs were determined using BET, which showed a slight decrease in the specific surface area for the annealed TiO_2_ NTs at 200 °C and *s* = 238.35 m^2^ g^–1^. The calcination as a postsynthesis treatment was employed to increase the crystallinity of the TiO_2_ NTs. Besides crystallinity, the size of crystallites also increased, while the specific surface area decreased. The structural stability of TiO_2_ NTs was altered as the calcination temperature increased. The decrease in the peak intensities and peak disappearance started at 300 °C due to the collapse of the NT structure and the loss of tubular integrity. As the calcination temperature increased to 400 °C, a phase transition was observed and surface area significantly decreased due to growth of TiO_2_ crystallites and the complete loss of the tubular integrity. The small decrease in the specific surface area of the TiO_2_ NTs at 200 °C could be explained by sintering damage until the calcination temperature was elevated enough to cause structural changes. The composition of the TiO_2_ NTs was also confirmed using FTIR. On the FTIR spectra, the characteristic bands of protonated titanate were observed as well as a broad band that corresponds to coordinated water molecules (see Figure S1 in the Supporting Information). Meanwhile, the AFM image of the sample showed agglomerates of nanotubes that formed after spreading the solution with TiO_2_ NTs on top of the mica sheet (see Figure 2b). Sizes of the TiO_2_ NTs were determined from the line profile shown in Figure S2a,c in the Supporting Information. The height was in the range from 5 to 15 nm and the length was in the range from 50 to 250 nm.

**Figure 3:**
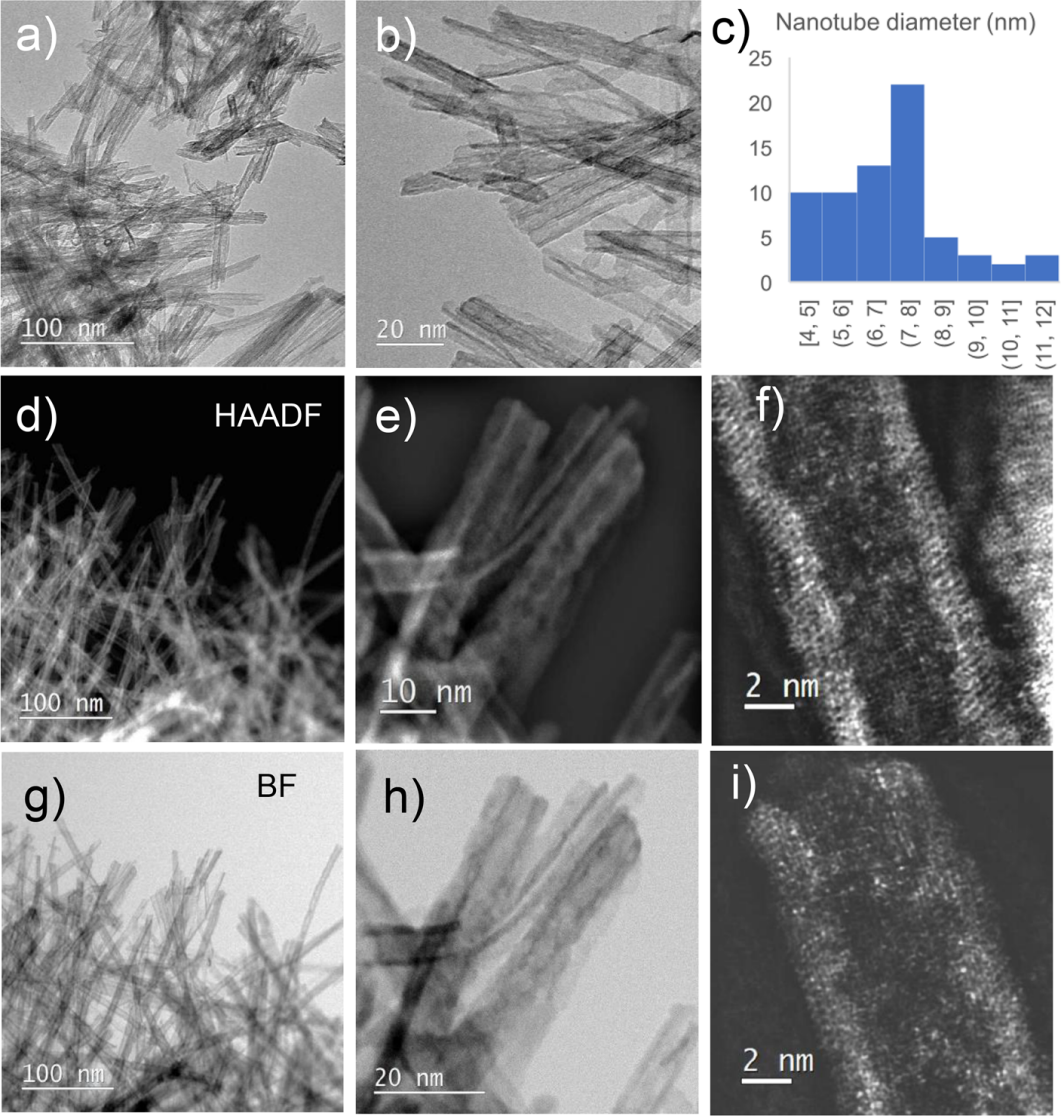
(a, b) TEM of TiO_2_ NTs sample. (c) Histogram of TiO_2_ NTs inner and outer diameter distribution. (d, f, i) STEM/HAADF micrograph of TiO_2_ NTs sample. (g, h) STEM/BF micrograph of TiO_2_ NTs sample.

The effect of the annealing process on the tubular morphology was also studied by using HR-TEM, where the powder sample was applied directly to a lacey carbon-coated Cu grid. The images of the nanoparticles show a characteristic tubular geometry contrast, in which the nanotubes walls display a higher intensity (darker in TEM and STEM-BF images and brighter in STEM-HAADF images, see Figure 3). During the annealing of the solid sample, the increase of the temperature from 200 to 500 °C resulted in the disappearance of the characteristic diffraction peaks of protonated titanate, while the anatase crystalline phase became dominant. To obtain the best crystallinity of TiO_2_ NTs, the temperature of 200 °C was chosen for the annealing process, to avoid any disruption of the tubular morphology (see Figure 2 in Supporting Information). TEM and STEM images are presented in Figure 3.

The length of the TiO_2_ NTs is around 200 nm on average; the histogram of inner and outer diameter distribution (Figure 3c) suggests that the inner radii *R*_inner_ range from 2 to 5.5 nm, with the most frequent value being *R*_inner_ = 3.5 nm while the outer radii *R*_outer_ range from 2.5 to 6 nm, with the most frequent value being *R*_inner_ = 4 nm. The thickness of the solid TiO_2_ layer (the thickness of the nanotube wall) is, on average, 1 nm. The contrasts in the TEM and STEM images in Figure 3 are characteristic of tubular geometry. The solid TiO_2_ NTs sample that was removed after the batch adsorption experiment was conducted (see the following section) was analyzed in order to identify the adsorbed Cs atoms. Since the intensity of the signal in STEM-HAADF images depends upon the atomic number, the bright spots in images f and i are individual Cs atoms.

### 4.2 Properties of TiO_2_ NTs Aqueous Suspensions

The properties of the aqueous suspensions of TiO_2_ NTs were probed by electrophoretic mobility measurements and by potentiometric acid–base titrations. The electrophoretic mobility measurements were conducted at two concentrations of CsNO_3_ in the system, namely, *c*(CsNO_3_) = 0.001 mol dm^–3^ and *c*(CsNO_3_) = 0.01 mol dm^–3^ (see Figure S4 in the Supporting Information). The results show the usual property of TiO_2_ NTs, which is a pronounced shift of the isoelectric point (pH_iep_) from pH = 3 at *c*(CsNO_3_) = 0.001 mol dm^–3^ to pH = 4 at *c*(CsNO_3_) = 0.01 mol dm^–3^.^23^ For pH values below pH_iep_, the TiO_2_ NTs are positively charged, while, for pH values above pH_iep_, the TiO_2_ NTs are negatively charged. Qualitatively, the shift in pH_iep_ with the increase of CsNO_3_ salt suggests specific cation partitioning in the vicinity of the nanotube (either adsorbed and/or in the EDL).^20^ Furthermore, the magnitude of the ζ potential is globally smaller in absolute value in the case of a higher CsNO_3_ concentration for the entire pH range, suggesting further screening of the surface charge.

**Figure 4:**
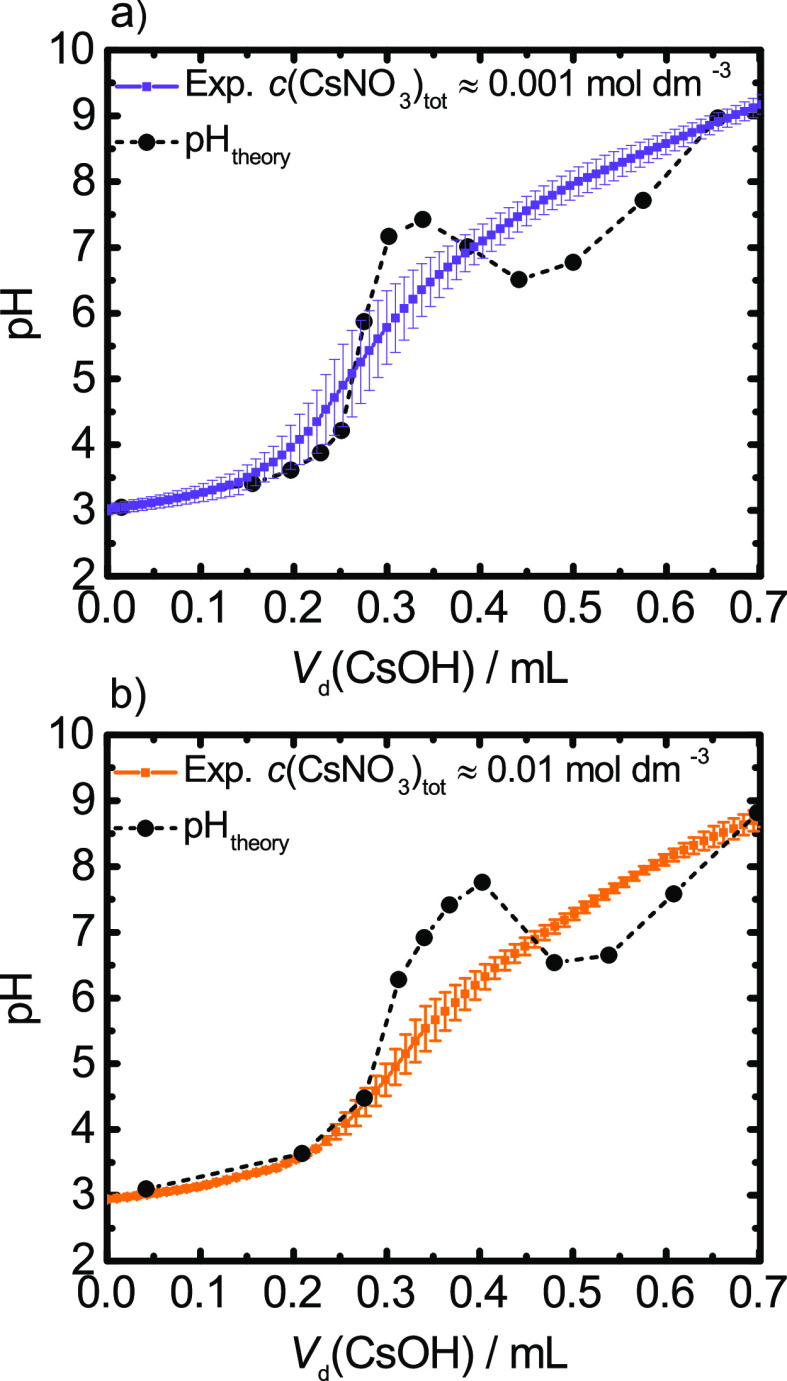
Calculated and measured pH with standard deviations in error bars of the aqueous suspension as a function of the added volume *V*_d_ of the base CsOH for the total Cs^+^ concentration at the end-point of the titration: (a)  mol dm^–3^; (b)  mol dm^–3^. Calculations were performed for radii *R*_inner_ = 3.5 nm, *R*_outer_ = 4 nm, site density Γ = 2.2, and association constants log  *K*_H,1_ = 8.5, log  *K*_H,2_ = 4.8, log  *K*_Cs,1_ = 1.2, and log  *K*_Cs,2_ = −0.2.

For further investigation concerning the ion partitioning in the TiO_2_ NTs system, we focused on the complementary potentiometric acid–base titrations. Those latter were systematically carried out from acid to base direction. The titration included the addition of the titrant (≈ 0.1 mol dm^–3^ CsOH) to the 0.01 mol dm^–3^ CsNO_3_ aqueous suspension of TiO_2_ NTs at γ = 1 g dm^–3^ or to the pure aqueous suspension. The analyzed potentiometric titration data are presented in Figure 4 along with the predictions of the theoretical mesoscopic model. The raw and treated data of measured pH of the suspension as a function of added CsOH base are presented in Figure S5 in the Supporting Information. Results of both  mol dm^–3^ and  mol dm^–3^ blank titrations show that there is not any clear inflection point, which suggests the presence of a contaminant that we identified as CO_2_. Titration curves of both TiO_2_ NTs suspensions overlap in the low and high additions of the CsOH base. The differences are visible in the intermediate region of the added base. At lower CsNO_3_ concentration ( mol dm^–3^), the measured pH of the suspension is systematically larger than for the higher CsNO_3_ concentration ( mol dm^–3^) in the intermediate region of the added base. This suggests the additional binding of Cs^+^ to the surface of TiO_2_ NTs and a related release of protons from the surfaces to the suspension (observed with lower pH). Note that the experimental values in the case of  mol dm^–3^ show lower standard error throughout the potentiometric acid–base titration experiment, compared to the  mol dm^–3^ case. This is due to the more efficient screening of the nanotube surface charge at higher salt concentrations.

We have also performed the batch adsorption of Cs^+^ ions experiment to test other aspects of TiO_2_ NTs suspensions. Results are presented in Figure S6a–c. The obtained data confirm that the increase of  concentration leads to a lower saturation of TiO_2_ NTs surface sites available for Cs^+^ ions adsorption.

### 4.3 Comparison of the Mesoscopic Model and Experimental Potentiometric Acid–Base Titration Data

The model was fitted versus the titration experiment. The output of the calculations is the full speciation of the system as a function of the volume of added CsOH base, in the presence of CO_2_ as “contaminant”. By speciation we consider namely the equilibrium concentrations of all ’mobile’ (e.g., H^+^, Cs^+^, OH^–^, NO_3_^–^, CO_3_^–^, and CO_3_^2–^) and “interfacial” species (e.g., surface sites at both inner and outer surface).

The comparison between the model predictions with association constants that correspond to the best fit and the experimental data is presented in Figure 4. Calculations were performed for the most probable set of TiO_2_ NTs radii, namely, *R*_inner_ = 3.5 nm and *R*_outer_ = 4 nm, as determined by HR-TEM measurements (Figure 3). The best fit was obtained with the following set of association constants: log  *K*_H,1_ = 8.5, log  *K*_H,2_ = 4.8, log  *K*_Cs,1_ = 1.2, and log  *K*_Cs,2_ = −0.2 (see Figure S10 in the Supporting Information). The site density Γ was fitted to the value 2.2. The obtained set of parameters is in the range of the “usual” values for these kinds of materials.^38,39^ To test the effect of the polydispersity of nanotube radii and the resulting effect of the curvature of the electric field onto ion-surface association phenomena, we performed the fitting with *R*_inner_ = 4 nm and *R*_outer_ = 6 nm. This radii set is far less represented (see Figure 3c) but corresponds to reduced curvature compared to the *R*_inner_ = 3.5 nm and *R*_outer_ = 4 nm case. Results are presented in Figure S13 in the Supporting Information. Again, the fitting of the experimental provided almost the same set of constants. We can conclude that the effect of the curvature of the electric field on ion-surface association phenomena is small compared to the effect of the chemistry and the Gibbs energy of ion-site association.

The model was tested for the two cases of CsNO_3_ concentrations in the aqueous suspension. The idea was to probe the physical conditions of the decontamination applications that range from dilute to moderately concentrated suspensions with salts. Figure 4a presents the  mol dm^–3^ case. In the range from *V*_d_(CsOH) = 1.5 to 0.3 mL, the model predicts a slightly lower pH of the aqueous suspension than the experimentally measured, but the discrepancy is within the range of the experimental error. In the same interval, the experiments show the largest discrepancy from the mean value. This can be attributed to the efficiency of screening of the metal–oxide surface by cations included in the charge regulation model, H^+^ and Cs^+^ in this case. Since the concentration of Cs^+^ in the acidic region is low, the charge of nanotubes is controlled by H^+^ from mineral acid. The model predicts the depletion of excess H^+^ into the solution from the nanotube surface, which acts as the apparent increase in the pH of the system. This is due to the overestimation of the repulsion of cation by Poisson–Boltzmann since it is an ideal model with no excluded volume of ions considered (ions are point-like charges). In the region between 0.3 and 0.4 mL, the model predicts a higher pH than the experimentally observed while, from 0.4 to 0.6 mL, the calculated pH is lower than the experimentally measured. For values larger than *V*_d_(CsOH) = 0.6 mL, the model is in good agreement with the experiments.

**Figure 5:**
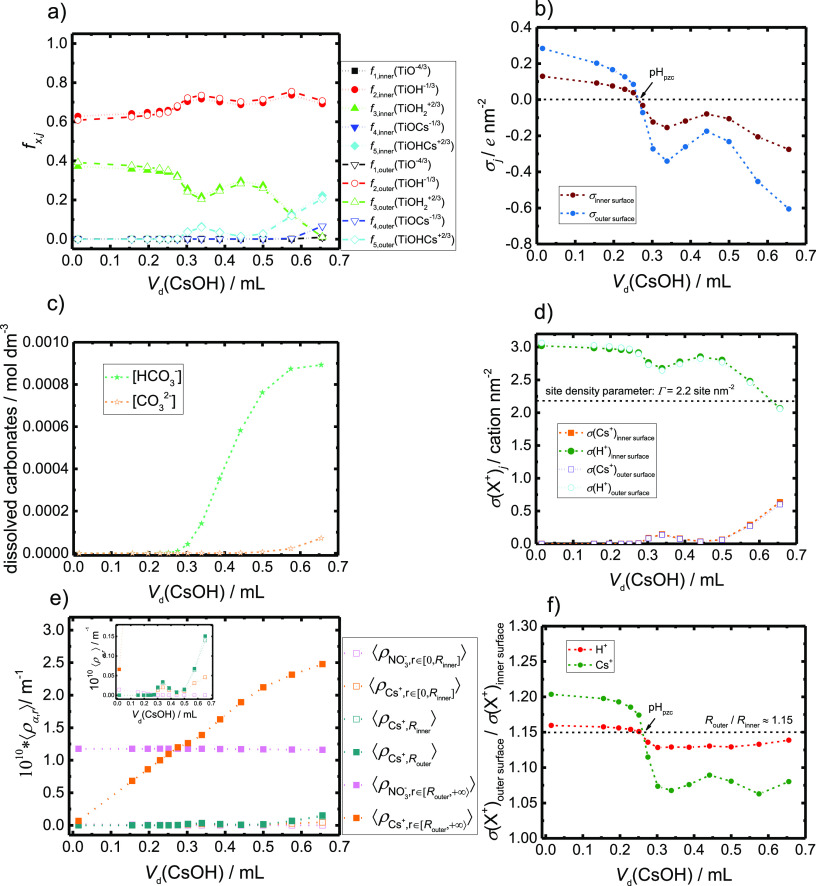
Full speciation of charged species within the titration experiment at *c*(CsNO_3_) = 0.001 mol dm^–3^. The figure shows (a) site populations, (b) surface charge densities, (c) carbonate species in aqueous solution, (d) surface cation coverage of surface active Cs^+^ and H^+^ cations, (e) volume integrals of Cs^+^ and NO_3_^–^ particle distribution functions normalized per unit length, and (f) ratio of surface cation coverage on both inner and outer interface, as a function of the volume of added base in the actual titration experiment. Calculations were performed for radii *R*_inner_ = 3.5 nm, *R*_outer_ = 4.0 nm, site density Γ = 2.2, and association constants log  *K*_H,1_ = 8.5, log  *K*_H,2_ = 4.8, log  *K*_Cs,1_ = 1.2, and log  *K*_Cs,2_ = −0.2.

Figure 4b presents the  mol dm^–3^ case. The agreement with experiments is better for lower and higher *V*_d_(CsOH) when compared to the  mol dm^–3^ case. Nevertheless, in the region between 0.3 and 0.4 mL, the model predicts higher pH values than the experimental observed values. The origin of this discrepancy can be attributed to the overestimation of the amount of dissolved CO_2_ by our model. The calculated equilibrium concentrations of HCO_3_^–^ and CO_3_^2–^ as a function of added CsOH are presented in Figure 5c.

### 4.4 Insight into Nanoscale Distribution of Charge within TiO_2_ NTs Systems in Aqueous Solutions

To understand the charge properties of the system, in addition to monitoring the pH of the suspension, we can plot the full speciation (sites and ions) as a function of *V*_d_(CsOH), for the whole titration experiment. The results are presented in Figure 5 for the  mol dm^–3^ case while the results for the  mol dm^–3^ case are presented in Figure S11 in the Supporting Information. The TiO_2_ NTs site populations (fraction of sites on the exposed surface *f*_*x*,*j*_; see eq 5) for the  mol dm^–3^ case are plotted in Figure 5a. First, overall, the differences in terms of surface chemistry on the inner and outer surfaces are small, even in low CsNO_3_ concentrations. In the acidic medium, at the beginning of the titration, the dominant surface sites are  and . As CsOH is gradually added in the suspension, the Cs^+^ concentration increases and the medium becomes more basic, the  site replaces . This process of Cs^+^-H^+^ competition for adsorption to the TiO_2_ NTs surfaces can be seen in Figure 5d), where the surface cation coverage (number concentration per unit surface) is plotted as a function of *V*_d_(CsOH). H^+^ dominates the inner and outer nanotube surfaces until there is a critical amount of Cs^+^ and OH^–^ ions in the system (close to millimolar concentrations), after which its surface concentration is decreased. This is the anticipated view of the process since protonation constants (log  *K*_H,1_ = 8.5 and log  *K*_H,2_ = 4.8) are much larger than Cs^+^ association constants (log  *K*_Cs,1_ = 1.2 and log  *K*_Cs,2_ = −0.2). The population of  site becomes significant only at highly basic pH, at the end of titration, i.e., at large *V*_d_(CsOH). Besides, in the case of a higher salt concentration in the system, i.e., for  mol dm^–3^, the site populations are radically changed (see Figure S11a in Supporting Information).  and  become more pronounced at lower *V*_d_(CsOH) due to the larger initial Cs^+^ concentrations while  populations are lowered.

The charge inhomogeneities in nanotube systems can be assessed by our model. With respect to the boundary conditions, we can divide the system into the interior of the nanotube, i.e. the inner surface, and the exterior of the nanotube, i.e. the outer surface. The average concentrations of Cs^+^ and  are therefore the volume integrals of the ions distributions, normalized by the unit length (we avoid dealing with edge effects). In the case of a higher salt concentration in the system, i.e.,  mol dm^–3^, the NO_3_^–^ average concentration is globally the same and the NO_3_^–^ ions (pink full squares) are predominantly outside the nanotube, toward the bulk. This result is in good agreement with the DFT-MD simulations presented in the following section. During the titration experiment, the addition of CsOH resulted in an accumulation of Cs^+^ ions in the system. The calculations demonstrate that the majority of the Cs^+^ ions are, actually, also present at the exterior of the NTs (orange full squares), but are predominantly in the EDL, i.e., close to the solid/liquid interface. A small fraction of the ions is present inside the NTs and also adsorbed to the inner and outer surfaces. In the case of a higher salt concentration in the system, i.e., for  mol dm^–3^, the significant observed changes include a higher Cs^+^ adsorption on the surface as well as Cs^+^ ions inside the NT and outside the outer surface (see Figure S11e in Supporting Information). Nevertheless, in both dilute  mol dm^–3^ and more concentrated cases  mol dm^–3^, the majority of the Cs^+^ cations in the system were not adsorbed to the surface.

Another important quantity that can be discussed is the surface charge density on the nanotube. During the titration experiment, the surface charge density is mostly affected by the pH and by the concentration of the ’surface active ions’, such as Cs^+^. In Figure 5b, the surface charge densities of the inner and outer surfaces σ_*j*_ are plotted as a function of *V*_d_(CsOH). The magnitude (the absolute value) of σ_outer surface_ is larger than σ_inner surface_ for the entire range of *V*_d_(CsOH), which is analogous for any pH. Both charge density curves present oscillations in the region between *V*_d_(CsOH) = 0.3 and 0.4 mL, which are visible in all graphs (a–d). This is not in agreement with the expected monotonous change with respect to the pH of the medium at equilibrium. These oscillations are just the result of the model for CO_2_ dissolution within Poisson–Boltzmann calculations, and there is no physical relevance.

To answer the question of the preferential cation adsorption between the inner and outer surfaces, we can plot the fraction of the cation surface coverage (data from Figure 5d). Results plotted in Figure 5f show that depending upon the pH (or *V*_d_(CsOH)), differences exist between the inner and outer NT surfaces. The ratio between the inner and outer radii (*R*_outer_/*R*_inner_ = 4/3.5) is around 1.15, and the fraction of the surface coverage for both H^+^ and Cs^+^ deviates from this value in a complex pH-dependent fashion. Furthermore, the deviations are more pronounced for the Cs^+^ cations. The aforementioned asymmetry of the distribution of cations between the inner and outer surfaces comes from the continuity of the potential expressed by eq 10 within the model. In the model, the dampening of the electric field (lowering the absolute value) as a response to the dielectric properties of the solid TiO_2_ causes the difference in electrostatic potential at the interfaces and affects the Boltzmann distributions. This was already reported in the literature.^29^ So far, this finding is purely based on theoretical grounds.

### 4.5 Molecular Level of Description of Cation Adsorption on TiO_2_: DFT-MD Simulations

#### 4.5.1 Hydration of the (101) Surface of TiO_2_

The effect of water adsorption on the (101) surface of TiO_2_ (see Figure S15a in the Supporting Information for the bare surface) was first assessed by filling the vacuum above the surface with water molecules. Two different input configurations were investigated:

- (1) All the water molecules were manually dissociated (on the two reciprocal surfaces), and the resulting hydroxy groups and protons were adsorbed on the five-coordinated surface titanium atoms and on the two-coordinated surface oxygen atoms, respectively;
- (2) All the water molecules were put in their molecular form, i.e., nondissociated.

Those two simulations were let evolve spontaneously and monitored over time (see Figure S15b,c and Figure S16a,b in Supporting Information). For the hydroxylated surface, some water molecules spontaneously reformed during the simulation, while for the nonhydroxylated surface, some water molecules spontaneously dissociated during the simulation. When a water molecule dissociated on the surface, the hydroxy group adsorbed on a five-coordinated surface titanium atom while the proton adsorbed on a two-coordinated surface oxygen atom (see Figure S15 in Supporting Information). This was in accordance with the experimental^66,67^ and theoretical works^53,68−70^ that all demonstrated the coexistence of molecular and dissociated forms of water molecules on the (101) surface of anatase. During the whole simulations, regardless of the input configuration, 100% of the five-coordinated surface titanium atoms were occupied either by an adsorbed hydroxy group or by a water molecule, which is typical of a classical hydrophilic surface.^71^ The average Ti–O bond length was (1.95 ± 0.05) Å in the case of hydroxy groups and (2.12 ± 0.05) Å in the case of nondissociated water molecules (see Figure S16c,d in Supporting Information). However, the two simulations did not converge to the same final configuration after 70 ps of simulation, which indicates that significant activation energy was probably required to attain the most stable configuration. This corresponded to an intermediate system between the two simulations, in which a part (20–50%) of the surface titanium atoms were occupied by hydroxy groups, i.e., by dissociated water molecules, and a part (50–80%) occupied by nondissociated water molecules. Consistently, the corresponding two-coordinated surface oxygen atoms were partly protonated. Overall, a dissociated water molecule corresponded to two ≡TiOH sites while a nondissociated water molecule corresponded to one ≡TiOH_2_ and one ≡TiO site, which is equivalent to the surface sites defined in eq 1 and 2. We can conclude that protonation equilibria can be safely modeled in a 2-p*K*_H,x_ manner within our mesoscopic model for the titration experiment.

#### 4.5.2 Adsorption of CsNO_3_ on the (101) Surface of TiO_2_

**Figure 6:**
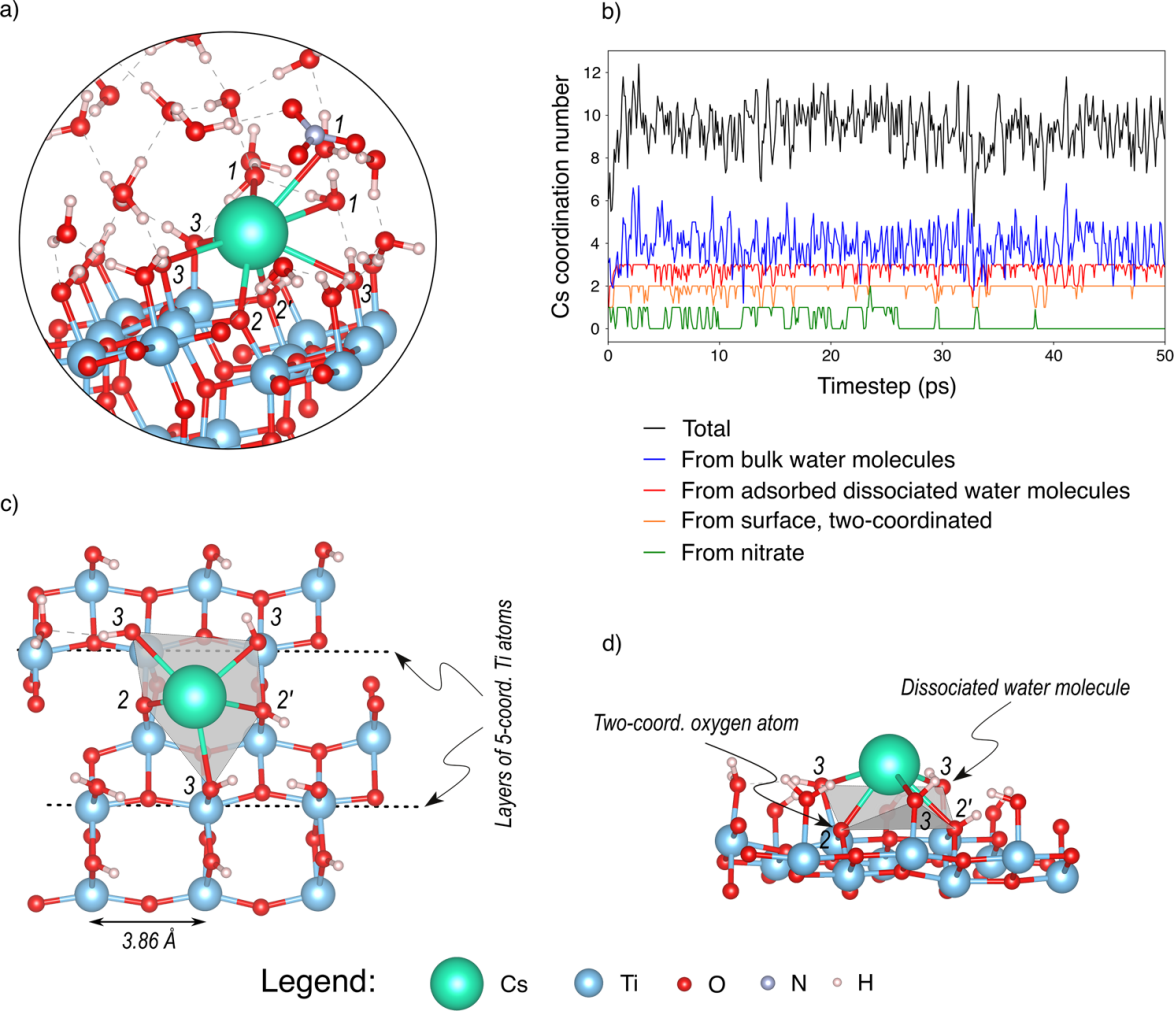
Results of the DFT-MD simulations. (a) A representative snapshot of the (101) surface of TiO_2_ seen from the side, with water and adsorbed Cs^+^ ion, showing the first coordination sphere of Cs^+^ ion composed of (1) bulk water molecules, (2) two-coordinated surface oxygen atoms, (2′) protonated two-coordinated surface oxygen atoms, and (3) dissociated adsorbed water molecules. (b) Evolution of the coordination of the Cs^+^ ion over simulation time (moving average on 200 fs), showing the different components of the Cs^+^ total coordination number. (c) Cs^+^ ion adsorbed on the TiO_2_ surface seen from the top, without representing the nonadsorbed water molecules. (d) The Cs^+^ ion adsorbed on the TiO_2_ surface seen from the side, without representing the nonadsorbed water molecules. In parts c and d, the gray zone represents the pentagon-like adsorption site, and the numbers are the same as in part a. In parts a–d, the dashed lines represent hydrogen bonds.

On a snapshot of the most stable hydrated system, i.e., with 30% of the surface Ti atoms hydroxylated, we added a CsNO_3_ molecule close to the surface by removing four water molecules in order to keep the volume constant. This avoided considering another thermalization period. Various input configurations for the orientation of the CsNO_3_ molecule regarding the surface plane were tested. We systematically observed the adsorption of the Cs^+^ ion on the TiO_2_ surface, while the nitrate ion did not adsorb and diffused to the water phase (see Figure S17 in Supporting Information), which was consistent with the assumptions we made in the Poisson–Boltzmann model. Besides, a first Bader charge analysis, performed on this system prior to the adsorption of Cs^+^, confirmed that the Cs atom was in the Cs^+^ form, while the nitrate group was in the NO_3_^–^ form. During the simulation, the Cs^+^ ion adsorbed on the (101) surface of TiO_2_ by establishing several Cs–O bonds with surface oxygen atoms as well as with several water molecules from the water phase (Figure 6a and Figure S18 in the Supporting Information for more detailed analysis of the Cs–O bond lengths). It exhibited a total coordination number comprised between eight and ten (Figure 6b), which was in very good agreement with the experimental and theoretical reported values.^72,73^ The number of water molecules comprised in the first coordination sphere and not adsorbed on the TiO_2_ surface (1 in Figure 6a) was comprised between 3 and 5, with an average value of 4. Besides, the nitrate ion, which was, at the beginning of the simulation, in the first coordination sphere of the Cs^+^ ion, progressively went away from the Cs^+^ ion and diffused to the water bulk (Figure 6b). The Cs^+^ ion established Cs–O bonds with two two-coordinated surface oxygen atoms (2 and 2’ in Figure 6a), i.e., the surface oxygen atoms that are undercoordinated, as well as with three surface hydroxy groups, which corresponded to dissociated water molecules adsorbed onto surface titanium atoms (3 in Figure 6a); see Figure 6c,d. The adsorption on two two-coordinated surface oxygen atoms and three adsorbed hydroxy groups is induced by the surface structure (Figure 6c,d): the three hydroxy groups are adsorbed on titanium surface atoms organized in layers in which they are separated by around 3.86 Å (Figure 6c). Two successive titanium atom layers are shifted of 1.93 Å from each other, which forms an adsorption cage composed of three adsorbed hydroxy groups and two two-coordinated surface oxygen atoms (represented in gray in Figure 6c,d). The two-coordinated surface oxygen atoms can be protonated or not, and the adsorbed hydroxy groups can also be nondissociated water molecules, considering the dynamic equilibrium of the surface in the presence of water. Therefore, the adsorption sites, represented by this five-coordinated cage, can exhibit a significant number of different chemical configurations. However, the adsorption of the Cs^+^ ion most probably induces the dissociation of the nondissociated water molecule on the three titanium atoms that surround the adsorption site and those are always in the ≡TiOH form. If we assume that no surface oxygen atoms are shared between adsorbed cesium ions when several Cs^+^ ions are added, the area of the effective adsorption site is around 80 Å^2^. This finding sets the requirement for the improvement of the mesoscopic model for definition of the surface site and complexation with larger monovalent ions such as Cs^+^.

A second Bader charge analysis, performed after the adsorption of the Cs^+^ ion, demonstrated a slight change in its atomic charges of +0.05 electrons, which can be attributed to the deformation of the electron cloud by the adsorption. It could be concluded that the adsorption of Cs^+^ on the (101) surface of TiO_2_ was electrostatic and, therefore, reversible. This is agreement with magnitude of Cs^+^ association constants that were determined by a mesoscopic model.

## 5 Discussion

In this study, we proposed a mesoscopic model to describe the charge properties and mass balance of the titration of metal–oxide nanomaterials that can be applied to different morphologies, such as spheres, rods, *etc*. There are a few key features that are increment to this study and need to be discussed in detail, such as the numerical stability of the code, the algorithm structure for solving highly nonlinear Poisson–Boltzmann equations, analytical description of chemical equilibria of all charged species in the system and their total balance, and spatial charge inhomogeneities for nanotubes system. From the aspect of the numerical implementation and solving the system of nonlinear Poisson–Boltzmann equations, the described algorithm showed numerical stability, i.e., generated reproducible results, for different salt concentrations and pH, ranging from dilute to more concentrated aqueous suspensions. Also, the results from the algorithm overcame the difficulty of evaluating the electrostatic potentials at the so-called ’high surface charge’ regime, for example, at high pH and salt concentration (see Figure 5b). Meanwhile, the algorithm satisfies the model condition of the Mass Action Law (semigrand canonical ensemble, where only water molecules are considered as the “reservoir”) for all the “mobile” ions and surface groups. This is, actually, a tool to probe and monitor concentrations of all ions, surface groups, and charge properties in a variety of system conditions involving metal–oxide nanomaterials. This is essential when describing experiments such as potentiometric acid–base titration where the ion concentrations change with every addition of the titrant. Additionally, by integrating a model for the estimation of the dissolution of CO_2_ and of the resulting carbonate protonation equilibria, it became clear that the pH of the dilute nanomaterials suspension is sensitive to time of the mixing or shaking time during the adsorption process.

From the analytical colloidal chemistry point of view, based on the practical example of potentiometric titration of TiO_2_ NTs with CsOH, the model predicted a moderate affinity of TiO_2_ NTs surface for Cs^+^ ions at acidic and neutral pH, due to the competition with H^+^. Even if the concentration of cesium ( mol dm^–3^) is increased by an order of magnitude in the aqueous solution, the surface charge properties of nanotubes are dominated by the protonation equilibrium. From the nanoscale description point of view, the proposed mesoscopic model can be used to probe the spatial average positioning of Cs^+^ cations (or any other ion) within the titration experiment. The results presented in Figure 5e and Figure S11 show that Cs^+^ cations are predominantly located in the electric double layer in contact with the outer nanotube surface, rather than in the interior or adsorbed on the surfaces. This spatial distribution of cations appears to be irrespective of the total cation concentration in the aqueous solution. This radical view of the adsorption phenomenon for aqueous suspensions of nanoporous metal oxide materials in thermodynamic equilibrium is completely opposite when batch experiments are identified as “the adsorption” (see Figure S6a–c in Supporting Information) suggesting even up to 50% of the total amount of Cs^+^ is adsorbed. In this equilibrium state of the dilute aqueous suspension, the nanomaterial is not an efficient adsorbent, as might be concluded when the definition of adsorption is based only on the batch experiments. In contrast to our findings, most studies so far suggest high adsorption efficiency, but there is a trick: most studies fit the adsorption data based on the batch experiments in which the solid adsorbent is removed from the suspension after the sufficient time (with assumed equilibrium achieved).^74^ It is worth emphasizing that the two states, the aqueous suspension at equilibrium and two-phase solid (adsorbent + adsorbate) and supernatant solution, correspond to completely different thermodynamic states. This is why the “adsorption efficiency” term should be used with great care.

The feature of the proposed model is a very important finding since it gives invaluable insight into the process at the nanoscale. When it comes to possible applications of nanotubes, most of which are aqueous suspensions (such as in catalysis or reversible decontamination), it is crucial to understand where ions are positioned in the system. This is not evident from batch adsorption experiments, which just show the “average” property of the system and, furthermore, reflect on the completely distinct thermodynamic states of two separated phases (solid and liquid).

Nevertheless, the model requires a few improvements. First, the prediction of the model that deviates from the monotonous change of suspension pH, site populations, and charging curves in the region between *V*_d_(CsOH) = 0.3 and 0.4 mL can be traced to the drawback of the modeled dissolution of CO_2_. In Figure 5c the concentrations of carbonate species HCO_3_^–^ and CO_3_^2–^ are plotted as a function of *V*_d_(CsOH). Results show an increase in the HCO_3_^–^ concentration after 0.3 mL of added CsOH, when the suspension approaches the neutral pH. CO_3_^2–^ species concentration increases under basic conditions, for which HCO_3_^–^ deprotonates (7). It should be noted that the precision of calculated carbonate concentrations is crucial precisely around the inflection point in the titration curve.

The predicted abrupt increase of HCO_3_^–^ in the region from 0.4 to 0.7 mL (pH from 6 to 8) is responsible for the increase of the proton concentration in the aqueous solution. As a consequence, our model which is in a canonical ensemble takes into account the additional proton concentration that evolves as HCO_3_^–^ is introduced by the dissolution of CO_2_.

At near- and pH-neutral system conditions, numerical solutions show an unexpected decrease in suspension pH, which is a consequence of HCO_3_^–^ deprotonation (eq 8). Note that this is not an error induced by the numerical noise of the code; rather, the results are reproducible with high confidence, where the absolute difference between iterative values of the Cs^+^, NO_3_^–^, H^+^, and OH^–^ ions concentrations set to 1 × 10^–8^ mol dm^–3^. The overestimated HCO_3_^–^ concentration is comparable to the site concentration. In the model, the deprotonation of HCO_3_^–^ has the dominant effect on the proton balance in the range from pH = 6 to 8.

It is worth noting, in a general fashion, that difficulty of the electroneutrality (such as at neutral pH, where H^+^ and OH^–^ concentrations are small) with the conjunction with any inaccurate estimate of the parameter (such as the dissolution of CO_2_ in our model) can lead to a significant discrepancy of the output of the calculation when compared to the experimental value.

Nevertheless, for practical applications, neutral pH is not often important, due to the reduced stability of metal oxide nanomaterials suspensions. Therefore, for practical use, the proposed model should already work sufficiently well, even for a quantitative estimate of ion adsorption.

Besides the inaccuracy caused by the estimate of CO_2_ dissolution within the model, we further explored the effect of the surface chemistry for the exposed (101) surface of TiO_2_ NTs. The aim was to test the approximation of the 1-site multiple association constants (p*K*_*x*_ for Cs^+^ and H^+^ associations) that we used for charge regulation of nanotubes. Association (adsorption) of Cs^+^ and H^+^ to the TiO_2_ NTs surfaces and the description of the “actual surface site” were studied in greater detail by molecular simulations. While for protonation, 1-site multiple associations constants can be justified to some extent,^38^ for Cs^+^ association, the approximation clearly does not hold. The size of the Cs^+^ ion and its first-sphere coordination shell cause binding to a few oxygen atoms on the titanate surface and in a complex structural modality. The generalization of the site model to account partitioning of multiple oxygen atoms in binding with Cs^+^ would be more adequate, and this is the area of our future work on the subject. Furthermore, in our mesoscopic model, we neglected co-ions (NO_3_^–^ and OH^–^ ions) specificity for the TiO_2_ NTs surfaces based on the argument of charge repulsion. The approximation was validated by simulations which showed that nitrate anion NO_3_^–^, even placed in the first coordination sphere of associated Cs^+^, diffuses toward the bulk aqueous solution. An important aspect of materials for water decontamination is their ability to be specific. In this study, we mainly looked at the competition between H^+^ and Cs^+^. There is a pH domain in which decontamination is possible in pure water for cesium ions, but this does not mean that it will be the case in any situation in real practical solutions, which can contain numerous metal ions. Nevertheless, the fact that adsorption of cesium appears to be reversible and that the Cs^+^ ion is adsorbed in well-defined surface states provides two interesting properties of the material. First, decontamination is based on weak interactions so that reversibility, which is important to regenerate the NTs, is possible with cesium. Second, the specific nature of adsorption sites for cesium ions makes the possibility of specific decontamination likely, but such a conclusion will need further developments.

## 6 Conclusions and Outlook

In this work, the integrative study based on experimental and theoretical approaches was applied to gain understanding in the adsorption of Cs^+^ on TiO_2_ NTs, which represented a case study for porous nanomaterials. TiO_2_ NTs were obtained by alkaline hydrothermal synthesis and underwent a postsynthesis calcination at 200 °C to improve their crystallinity. The morphological features of TiO_2_ NTs were characterized in detail via HR-TEM and AFM, which confirmed the tubular morphology. Most NTs presented a length of 200 nm, an inner radius of 3.5 nm, and outer radius of 4 nm. Acid–base surface properties of TiO_2_ were elucidated by electrophoretic measurements and potentiometric titrations. The results revealed that the isoelectric and point of zero charge shift, which suggests the adsorption of Cs^+^ cations onto TiO_2_ NTs. To gain insights into the distribution of the adsorbed Cs^+^ cation within TiO_2_ NTs (and nanoporous materials in general), we derived a mesoscopic model that can be used to quantitatively describe the full speciation of the system during the spam of the potentiometric acid–base titration of the metal-oxide nanomaterials. Furthermore, the model is derived such that it includes CO_2_ dissolved in the aqueous suspension. The goal was to propose a model that can predict the separation of metal ions, ranging from dilute to moderately concentrated 1:1 salt aqueous suspensions.^75^

The key findings state that H^+^ dominates in TiO_2_ NTs charging process, while Cs^+^ becomes important only at high concentrations and in basic pH region. Furthermore, CO_2_ is an important contributor to the equilibrium in the system and thus cannot be neglected. In the case of shorter nanotube radii (*R*_inner_ = 3.5 nm, *R*_outer_ = 4 nm), the inner and the outer surfaces of the nanotube present similar surface sites populations and ion–surface association equilibrium states. The curvature of the nanotube has a very small effect as long as the TiO_2_ NT solid layer is thin. The comparison between model predictions and results of the potentiometric acid–base titrations showed the discrepancy around neutral pH, which originates from the overestimation of the dissolved CO_2_ by our model. When used to deduce spatial distribution of ions and charges in the system, the model proposed the radical explanation that in the aqueous suspension the majority of the target solute Cs^+^ is in the electric double layer around the outer surface, rather than adsorbed at the surface of nanotubes. This finding is especially important to stress in the context of possible application in terms of catalysis or reversible separations, where it is essential to understand the spatial partitioning or more simply said: “where the target species are located”. Besides, atomistic *ab initio* MD simulations were performed to test the 1-site multiple ion association approximation within the mesoscopic model. The simulations showed that for protons and co-ions the model works sufficiently well, but for larger Cs^+^ cations, the definition of surface site is inadequate due to the size of the cation.

The authors are currently working on the extension of this study in terms of improvements of the charge regulation model of surface sites as well as integration of the kinetic estimate of CO_2_ from the ambient atmosphere. In parallel, we are trying to perform a stereological estimation of the surface coverage of Cs^+^ onto TiO_2_ NTs by counting bright dots within HAADF images after the adsorbent is removed from the mixture in batch experiments. We plan to “follow” the adsorption isotherm from very dilute to concentrated aqueous solutions. At the same time, the use of a derived mesoscopic model can give insight into adsorption efficiency before the adsorbent is removed from the mixture.

## Supporting Information Available

Synthesis of TiO_2_ NTs, annealing of TiO_2_ NTs sample, structural characterization of TiO_2_ NTs, properties of the aqueous suspension of TiO_2_ nanotubes, batch experiments of Cs^+^ adsorption on TiO_2_ NTs, full model derivation: Poisson–Boltzmann theory to reproduce acid–base titration curve of TiO_2_ NTs aqueous suspension in dilute regime with CO_2_ as a contaminant, fitting of the model to the experimental data, model predictions for different sets of inner and outer nanotubes radii, first-principles Molecular Dynamics Simulations (PDF)
