# Supplementary material for: Cation Adsorption in TiO2 Nanotubes: Implication for Water Decontamination
Source: ACS Appl Nano Mater. 2023 Jul 11;6(14):12711–25. doi: 10.1021/acsanm.3c00916 (PMC10391741; doi:10.1021/acsanm.3c00916)
Supplement: Supplementary file 1 — an3c00916_si_001.pdf [file an3c00916_si_001.pdf]

Supporting Information: Cation Adsorption in TiO_2 Nanotubes: Implication for Water Decontamination

Atiđa Selmani[a,g], Bertrand Siboulet[c], Mario Špadina*[a,b], Yann Foucaud[c], Borna Radatović[d], Karla Korade[f], Ivan Nemet[f], Goran Dražić[e], Davor Kovačević[f], Jean-François Dufrêche*[c], and Klemen Bohinc*[b]

[a]Ruđer Bošković Institute, Division of Physical Chemistry, 10000 Zagreb, Croatia
[b]Faculty of Health Sciences,University of Ljubljana, Zdravstvena 5, SI-1000 Ljubljana, Slovenia
[c]ICSM, Univ Montpellier, CEA, CNRS, ENSCM, Marcoule, France
[d]Institute of Physics, Bijenička 46, 10 000 Zagreb, Croatia
[e]National Institute of Chemistry, Laboratory for Materials Chemistry, SI-1000 Ljubljana, Slovenia
[f]Faculty of Science,University of Zagreb, Horvatovac 102A, 10 000 Zagreb, Croatia
[g]Institute of Pharmaceutical Sciences, Pharmaceutical Technology & Biopharmacy, A-8010 Graz, Austria

Contents

1 Synthesis of TiO_2 NTs S-2

2 Annealing of TiO_2 NTs sample S-2

3 Structural characterization of TiO_2 NTs S-3
3.1 The powder *X*-ray diffraction (PXRD) S-3
3.2 Attenuated total reflectance Fourier transform infrared spectroscopy (ATR FTIR) S-3
3.3 The specific surface area *via* Brunauer-Emmett-Teller method S-3
3.4 Atomic force microscopy (AFM) S-4
3.5 High-resolution transmission electron microscopy (HR-TEM) S-4

4 Properties of the aqueous suspension of TiO_2 nanotubes S-4
4.1 Preparing the TiO_2 NTs suspension - testing the optimal sonication procedure S-4
4.2 Electrophoretic measurements S-5
4.3 Potentiometric acid-base titrations S-6
4.4 Batch adsorption experiments - Cs uptake onto nanotubes S-7

5 Model based on Poisson-Boltzmann and charge regulation theories to reproduce acid-base titration curve of TiO_2 NTs aqueous suspension in dilute regime with CO_2 as contaminant S-8
5.1 Charge regulation of the surface charge density: Mass Action Law and local concentrations of ions S-8
5.2 Electrostatics of the system through Poisson-Boltzmann calculations S-10
5.2.1 Validity of cell model through experimental argument S-10
5.2.2 Free energy functional S-11
5.3 Correction due to dissolution of CO_2 within titration experiment S-12
5.4 Concentration of all ions within the titration experiment S-13
5.4.1 Practical example: the titration with CsOH titrant that starts from the acidic medium at a given $CsNO_3$ concentration S-13
5.5 Fitting the blank acid-base titration to obtain $[CO_2]^{tot}_{aq}$ S-14
5.5.1 Obtaining CO_3^{2-}, and HCO_3^- speciation in the TiO_2 NTs suspension S-14
5.6 Calculation of Cs^+ distributions from P.B. in the aqueous suspension of TiO_2 NTs S-15
5.7 General relation between V_B and $[Cs^+]^{total}$, *i.e.*, how to connect the model and the experiments S-16

5.8 Numerical procedure: implementing the model S-17
5.8.1 Program flowchart S-17

6 **Model predictions** **S-18**
6.1 Fitting of the model S-18
6.2 Full speciation of all charged species in the system for $c(CsNO_3) = 0.01$ mol dm^{-3} S-18
6.3 Surface TiO_2 NTs properties and speciation for larger radii: $R_{inner} = 4$ nm, $R_{outer} = 6$ nm . S-19
6.3.1 Dissimilarities between the inner and outer surfaces charging and cation adsorption S-19

7 **First-principles Molecular Dynamics Simulations** **S-23**
7.1 Computational Details S-23
7.2 Structural Model S-23
7.3 Results S-25
7.3.1 Hydration of the (101) surface of TiO_2 . . . . . S-25
7.3.2 Adsorption of $CsNO_3$ on the (101) surface of TiO_2 . . . . . S-26

1 Synthesis of TiO_2 NTs

TiO_2 nanotubes (TiO_2 NTs) were synthesized using a hydrothermal synthesis route similar to that described by Kasuga *et al.*[1] and in our previous work[2,3]. 2 g of anatase TiO_2 nanoparticles were dispersed into 65 mL of a 10 mol dm^{-3} sodium hydroxide solution. The homogenous dispersion was achieved by ultrasound that was applied for 1 hour at 25 °C. The anatase TiO_2 dispersion was placed in a PTFE-lined autoclave and heated for 65 hours at 140 °C. The white precipitate produced after the synthesis was washed thoroughly with ultra-pure water (resistivity of 18.2 MΩ) to remove the excess sodium hydroxide and other impurities that remained after the synthesis. The washing treatment was repeated until the pH and conductivity of the supernatant were close to the values for the ultra-pure water. The washed precipitate was incubated with 0.1 mol dm^{-3} hydrochloric acid for 3 hours to remove excess of adsorbed sodium ions. The final product was washed extensively with deionized water to remove acidic impurities until the conductivity and pH of the supernatant were close to the pH and conductivity of ultra-pure water. The TiO_2 NTs were dried at 80 °C for 6 h in air and stored in glass bottles in the dark.

2 Annealing of TiO_2 NTs sample

Reported studies on 'purifying' TiO_2 NTs from amorphous TiO_2 phase by high temperature annealing suggest that the fraction of the amorphous phase decreases around 300 °C at longer heating times, and anatase phase becomes dominant. The reported experimental conditions (the temperature) for the removal of amorphous TiO_2 strongly depend on the synthesis procedure[4]. Therefore, we conducted our independent study for our specific TiO_2 NTs sample. The procedure included high temperature annealing of the sample in the furnace (Chamber Furnace up to 1400 °C, Nabertherm GmbH, Germany). The duration of the annealing process was 3 h, followed by PXRD and HR-TEM analysis. As the temperature increased from 200 to 500 °C, the characteristic diffraction peaks of protonated titanate disappeared, and anatase crystalline phase predominated. After 400 °C the phase transition occurred and particles underwent from tubular to solid random shaped structure. In an iterative fashion, we reached optimal conditions for removing most of the excess amorphous phase at 200 °C, while retaining the nanotubuluar morphology. The resulting sample had nanoparticles with tubular layered structure and anatase planes were identified.

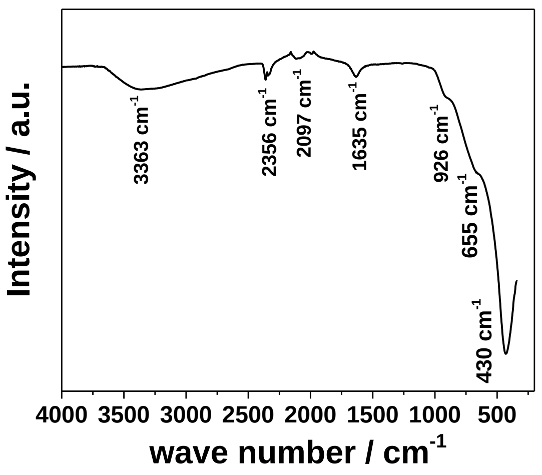

Fig. S1 ATR FTIR spectra for raw TiO_2 NTs

3 Structural characterization of TiO_2 NTs

3.1 The powder X-ray diffraction (PXRD)

The structural characterization of raw and annealed TiO_2 NTs was performed by powder X-ray diffraction (PXRD). PXRD data were collected by Aeris Panalytical diffractometer with Ni-filtered copper radiation, in Bragg-Brentano geometry with the sample prepared in a thin layer on a silicon zero-background holder. PXRD patterns were scanned in the range $2\theta = 5$ - $50°$ with a step size of 0.005° and 10 s per step. The Panalytical High Score Plus software suite was used for data treatment.

Obtained diffraction patterns of TiO_2 NTs at 2θ degree: 10.4, 24.5, 28.59 and 48.5 correspond to typical PXRD pattern of protonated titanate ($H_2Ti_3O_7$). The observed diffraction patterns are in good agreement with those published previously[5,6].

3.2 Attenuated total reflectance Fourier transform infrared spectroscopy (ATR FTIR)

Attenuated total reflectance Fourier transform infrared spectroscopy (ATR FTIR) measurements were carried out on an FTIR spectrometer equipped with an attenuated total reflection module (Tensor I, Bruker, Ettlingen, Germany) from 4000 – 400 cm^{-1}. The step of the ATR FTIR spectrophotometer was 2 cm^{-1}.

Results are presented in Figure 1 The bands at 430, 656, 926, 1635, 1984, 2356 and 3347 cm^{-1} confirmed a good agreement with previously published data[7,8].

3.3 The specific surface area *via* Brunauer-Emmett-Teller method

The specific surface area, s, of TiO_2 NTs was determined employing the multipoint Brunauer-Emmett-Teller method using N_2 as adsorbed gas at 77 K and relative pressure values in the range 0.05-0.3 on a Micrometrics Instrument Corporation, Gemini V series Surface Area Analyzer. Obtained s values were:

- 1) 260.72 $m^2\ g^{-1}$ for the raw TiO_2 NTs,
- 2) 238.35 $m^2\ g^{-1}$ for TiO_2 NTs sample annealed at 200 °C.

3.4 Atomic force microscopy (AFM)

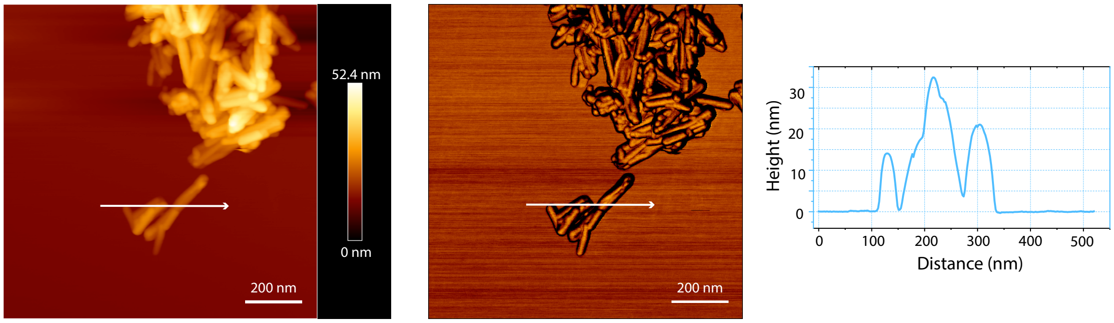

Fig. S2 a) Topography (height) image of annealed TiO2 NTs sample. b) Phase image of the same region. c) Line profile taken along a white arrow on a).

Atomic force microscope (AFM) images of TiO_2 NTs were taken with JPK Nanowizard Ultra Speed AFM under ambient conditions. A small amount of TiO_2 NTs suspension, 20 μL was deposited on an atomically flat mica sheet and left to dry for one hour before measurements. Non-contact AC (tapping) mode was used for acquisition with a setpoint of 52%. AppNano silicon tips with a nominal spring constant of 58 N m^{-1}, a tip radius less than 5 nm and a nominal resonant frequency of 190 kHz were used. Images were processed with JPK Data Processing software.

Figure 2 represents AFM image of the agglomerate of nanotubes which formed after spreading suspension with TiO_2 NTs nanotubes on top of mica sheet. From the line profile shown on 2c, which is taken from the area highlighted with the white arrow, the shape and size of individual nanotubes can be determined. Line profile display single, triple and double nanotube which can be verified from phase image 2b. The height of individual nanotubes is around 10 nm and the length in ranges from 50 nm to 250 nm.

3.5 High-resolution transmission electron microscopy (HR-TEM)

Probe Cs-corrected scanning transmission electron microscope (STEM), model Jeol ARM 200 CF, operated at 80 kV (to minimise the electron-beam related damage to the material), coupled with Gatan Quantum ER Electron Energy Loss Spectroscopy system and energy dispersive x-ray spectrometry (Jeol Centurio 100) was used to study the structure and chemical composition. The powder sample was applied directly to a lacey carbon-coated Cu TEM grid. The images of the nanoparticles show a characteristic tubular geometry contrast, where the nanotube walls show higher intensity (darker in TEM and STEM - BF images and brighter in STEM - HAADF images). In the atomic-resolution STEM - HAADF images, the bright spots are individual Cs atoms, since the signal intensity in these images is related to the atomic number (the atomic number of Ti is 22 and that of Cs is 55).

4 Properties of the aqueous suspension of TiO_2 nanotubes

4.1 Preparing the TiO_2 NTs suspension - testing the optimal sonication procedure

The aim of the procedure is to achieve homogeneous suspension of TiO_2 NTs, while minimizing the breakage of nanotubes. Therefore TiO_2 NTs suspensions were subjected to two different types of sonication treatment: 1) sonication in the ultrasound bath, and 2) application of the probe-type ultrasonic homogenizer. For the sonication in the ultrasound bath (35 kHz, 320 W, Bandelin Sonorex Rk 100 H) TiO_2 NTs suspensions were sonicated in the following time periods: 15, 30, 45 and 60 minutes. Since

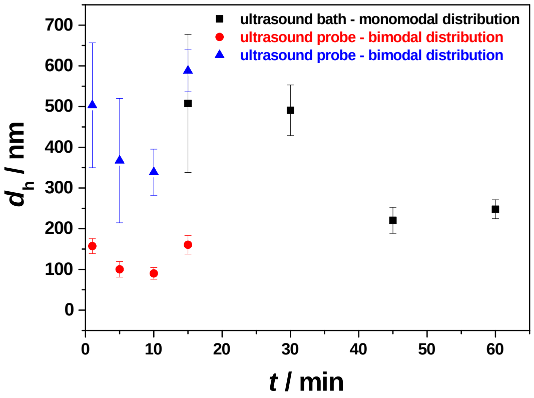

Fig. S3 The variation of hydrodynamic diameter (d_{h}) with the sonication time of annealed TiO_2 NTs. Black squares correspond to sonication with ultrasound bath, while red and blue triangles correspond to the ultrasound probe.

the probe-type ultrasonic homogenizer (1500 V, 20 kHz, Sonicator Ultrasonic Processor XL, Misonix Inc.) provides stronger sonication than the ultrasound bath, the TiO_2 NTs were treated for 1, 5, 10 and 15 mins. Dynamic Light Scattering (DLS) for particle sizing and distribution was determined by means of Brookhaven 90Plus/BI-MAS. Results are presented in Figure S3. Results show bimodal distribution of d_{h} when probe-type ultrasonic homogenizer was applied.

4.2 Electrophoretic measurements

Zeta potential measurements of TiO_2 NTs were determined by means of ZetaPlus, Zeta potential Analyzer, Brookhaven 90Plus/BI-MAS. For the electrokinetic measurements TiO_2 NTs suspensions, $\gamma(\mathrm{TiO_2}) = 0.05$ g dm^{-3}, were prepared at $CsNO_3$ concentrations, $c(\mathrm{CsNO_3}) = 0.001$ mol dm^{-3} and $c(\mathrm{CsNO_3}) = 0.01$ mol dm^{-3}. Initial pH of TiO_2 NT suspension pH, was adjusted with 0.1 mol dm^{-3} CsOH. Suspensions were sonicated for the 5 min using the ultrasound horn. Both TiO_2 NT suspensions ($c(\mathrm{CsNO_3}) = 0.001$ mol dm^{-3} and $c(\mathrm{CsNO_3}) = 0.01$ mol dm^{-3}) were titrated with 0.1 mol dm^{-3} HNO_3 under magnetic stirring to prevent sedimentation. The equilibration time between each titrant addition was 5 minutes. During that time interval the stable pH electrode signal was obtained with the potentiometer accuracy of 0.01 mV. The experiments were repeated at least 2 times. Results are presented in Figure 4.

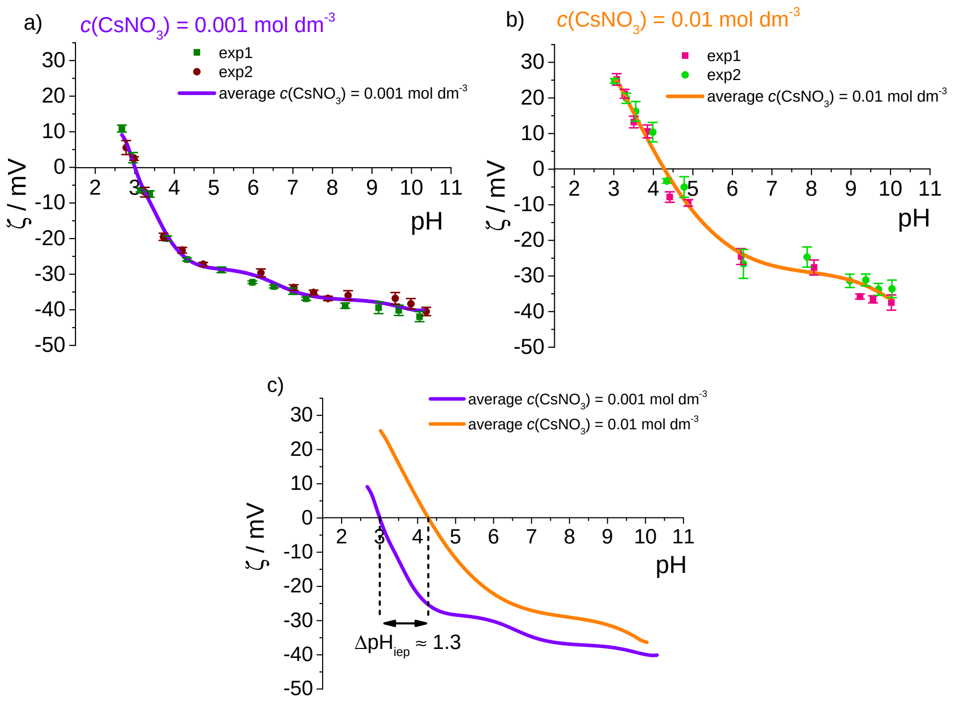

Fig. S4 Electrophoretic mobility of TiO_2 NTs expressed through ζ potential as a function of pH for: a) $c(\mathrm{CsNO3})_{\mathrm{aq,eq}} = 0.001$ mol dm^{-3}, b) $c(\mathrm{CsNO3})_{\mathrm{aq,eq}} = 0.01$ mol dm^{-3}, c) calculated average curves for both cases with depicted shift in $\mathrm{pH_{iep}}$. In both experiments mass density of TiO_2 NTs suspension was $\gamma(\mathrm{TiO_2}) = 0.05$ g dm^{-3}.

4.3 Potentiometric acid-base titrations

The titration setup was composed of a pH meter (826 pH, Metrohm) equipped with combined electrode (Metrohm, 6.0234.100) and a thermostatic titration cell regulated at 25 °C by a thermocryostat (Julabo F12). Electrode was calibrated with five standard buffers (Riedel-de Haen). CsOH was standardized by potentiometric titration with KHP. This solution was then used to determine HNO_3 concentration. Deionized and CO_2 free water was used for all experiments. The CO_2 free water was obtained by heating deionized water to the boiling point, followed by a passage of inert gas during 6 h. This ensured no CO_2 was dissolved prior to the titration experiment. Suspensions were sonicated for the 5 min using the ultrasound horn. The potentiometric acid-base titrations were carried out from acid to base direction. Titration included the addition of the titrant ($\approx$ 0.1 mol dm^{-3} CsOH) either to the background electrolyte (10^{-3} or 10^{-2} mol dm^{-3} $CsNO_3$) or to the aqueous suspension of TiO_2 NTs at $\gamma = 1$ g dm^{-3} and equilibration to a potential drift rate (monitored every 4 s) of less than 0.05 mV min^{-1}. Once a stable potential was attained, pH was recorded. V_b is volume of blank titration and V_a is the volume in suspension. Potentiometric titration was repeated 4 times for each initial concentration of $CsNO_3$.

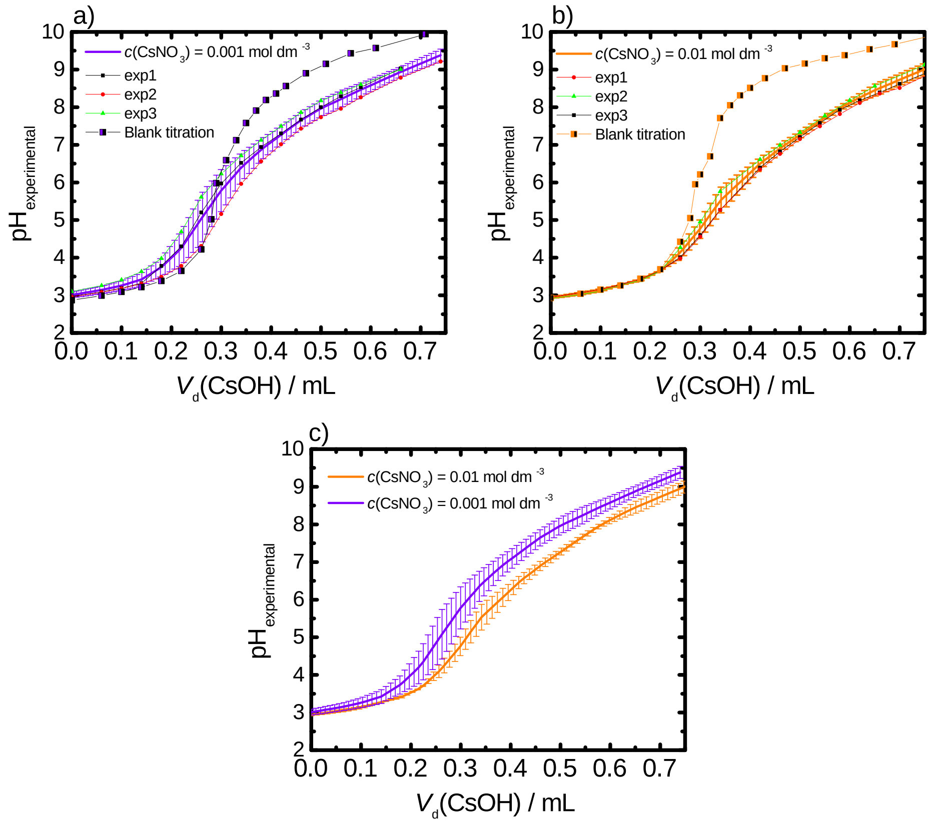

Fig. S5 Potentiometric acid-base titrations TiO_2 NTs: measured pH as a function of the volume of added base CsOH V_d. Solid two-colored squares depict blank titrations. For both $CsNO_3$ concentrations, the titrations of TiO_2 NTs suspensions were repeated 3 times. Raw data are depicted as symbols interpolated with straight line, while the average of the 3 curves are presented with error bars that reflect standard deviation. At the end point of the titration experiment the total concentration of Cs^+ in the system is: a) $c(\mathrm{Cs}^+)_{\mathrm{aq,eq}} = 0.001$ mol dm^{-3}, b) $c(\mathrm{Cs}^+)_{\mathrm{aq,eq}} = 0.01$ mol dm^{-3}, c) comparison of average values and standard deviations in error bars from titrated suspension in a) and b).

4.4 Batch adsorption experiments - Cs uptake onto nanotubes

For the determination of cesium concentration a Teledyne Leeman Labs. (Hudson, NH, USA) Prodigy High Dispersion ICP system was used. The instrument is equipped with 40 MHz "free-running" radiofrequency generator and an echelle grating spectrometer with a large-format programmable array detector (L-PAD). The generator power of 1.1 kW and flow rates of argon (coolant 18 L min^{-1}, auxiliary 0.8 L min^{-1}) was held constant in all experiments. Emission lines of cesium at 894.347 nm were selected from the image on the detector as the line without background and spectral interference. Additional purging of spectrometer optics with argon was switched on during signal acquisition. Integration time was adjusted to 10 s and signal acquisition at cesium line was repeated three times for each measurement. The precision of intensity measurements on chosen analytical emission line was 0.4 - 2.5% RSD. Single element standard solution of Cs CGCS1-1 (Inorganic Ventures, 300 Technology Drive, Christiansburg, VA 24073) was used for control of plasma line positioning and the preparation of calibration standard solutions. In order to verify the accuracy of the analytical procedure certified reference material of argillaceous limestone (NIST SRM 1d) was used. This CRM was chosen as the most similar to the analyzed samples matrix with declared cesium content of 400 μg kg^{-1}. The mea-

sured concentration of cesium on chosen emission line comprised 392 μg kg^{-1}, which yield a recovery value of 98%. All the samples and reference samples were analyzed as triplicate. Blanks were also prepared in a manner identical to that of samples and were measured before the set of analytical samples. Results are presented in Figure 6. The obtained data confirm that the increase of $c(\mathrm{CsNO_3})_{\mathrm{tot}}$ concentration leads to a lower saturation of TiO_2 NTs surface sites available for Cs^+ ions adsorption.

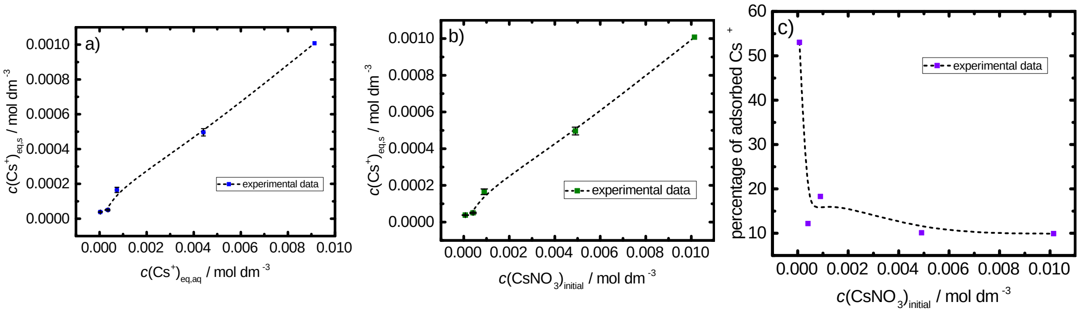

Fig. S6 Batch experiment determination of adsorbed Cesium onto TiO_2 NTs. Batch adsorption was conducted under air atmosphere and pH was initially 7. For all $CsNO_3$ concentrations, the experiments were repeated 2 times. Adsorption data are depicted as symbols with error bars that reflect standard deviation. Dashed line are given as guideline. a) $c(\mathrm{Cs^+})_{\mathrm{eq,s}}$ as a function of $c(\mathrm{Cs^+})_{\mathrm{eq,aq}}$, b) $c(\mathrm{Cs^+})_{\mathrm{eq,s}}$ as a function of $c(\mathrm{CsNO_3})_{\mathrm{initial}}$, c) percentage of adsorbed cesium as a function of $c(\mathrm{CsNO_3})_{\mathrm{initial}}$. Percentage of adsorption cesium is defined[9] as $(\%) = 100\% \times (c(\mathrm{CsNO_3})_{\mathrm{initial}}$ - $c(\mathrm{Cs^+})_{\mathrm{eq,aq}})/c(\mathrm{CsNO_3})_{\mathrm{initial}}$

5 Model based on Poisson-Boltzmann and charge regulation theories to reproduce acid-base titration curve of TiO_2 NTs aqueous suspension in dilute regime with CO_2 as contaminant

5.1 Charge regulation of the surface charge density: Mass Action Law and local concentrations of ions

To represent the aqueous suspension of TiO_2 NTs, we consider an infinitely long TiO_2 NT immersed in the aqueous solution of ions. Due to the experimental condition of low suspension mass density ($\gamma = 1$ g dm^{-3}) and moderate $CsNO_3$ concentrations ($c(\mathrm{CsNO_3})$ from 0.001 mol dm^{-3} to 0.01 mol dm^{-3}), we neglect interactions between TiO_2 NT. Nanotube surface charge is modulated *via* a charge regulation mechanism[10]. Figure S7 shows the schematic representation of the charging processes that take place at both TiO_2 NT surfaces. Both surfaces have the same site density, and the same type of sites and are in contact with the aqueous solution of ions. The dielectric properties of the aqueous solution $\varepsilon_{\mathrm{r},1}$, and solid TiO_2 $\varepsilon_{\mathrm{r},2}$ are considered equal ($\varepsilon_{\mathrm{r},1} = \varepsilon_{\mathrm{r},2} = 80.1$). We consider anatase planes based on structural data (see section Results). Due to the heterogeneity of the TiO_2 NTs samples[11] (amorphous solid, differently exposed atoms, polydispersity of sizes, *etc.*), we simplified the model and thus avoided dealing with numerous number of parameters[12]. Rather we focus on the phenomenon of preferential cation adsorption between exposed surfaces. For the adsorption sites, we consider a one-site, multiple equilibria model for successive protonations and Cs^+ associations onto the exposed oxygen atom[13].

We do not consider NO_3^- association to TiO_2 NTs surfaces. Based on performed *ab initio* MD simulations it was shown that nitrate is repelled from the anatase surface and diffuses towards a bulk aqueous solution (see Figure S17). The charges of exposed single-boned oxygen atoms are estimated by Pauling valence concept[14,15]. We obtained the following set of equilibria, expressed as site-ion associations constants:

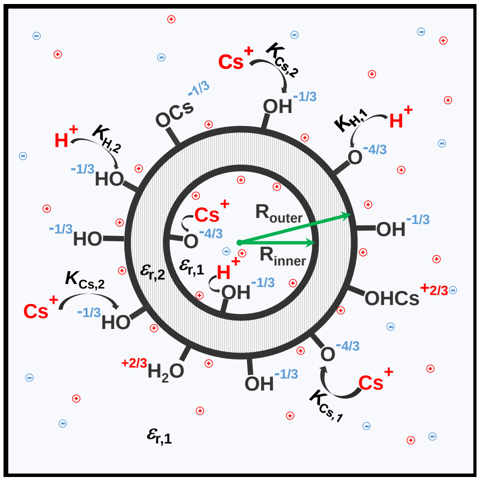

Fig. S7 Schematic cross-section of TiO_2 NTs. The cross-section is perpendicular to the cylinder axis of symmetry. R_{inner} and R_{outer} respectively correspond to the inner and the outer cylinder radii. $\varepsilon_{\mathrm{r},1}$ and $\varepsilon_{\mathrm{r},2}$ correspond to dielectric constants of the electrolyte solution and the solid TiO_2 NT, respectively. Surface groups $\equiv \mathrm{TiO}^{-\frac{4}{3}}$, $\equiv \mathrm{TiOH}^{-\frac{1}{3}}$, $\equiv \mathrm{TiOCs}^{-\frac{1}{3}}$, $\equiv \mathrm{TiOH}_2^{+\frac{2}{3}}$, and $\equiv \mathrm{TiOHCs}^{+\frac{2}{3}}$ are in equilibrium with a surrounding electrolyte solution. $K_{\mathrm{H},1}$ and $K_{\mathrm{H},2}$ are successive surface protonation reactions, while $K_{\mathrm{Cs},1}$ and $K_{\mathrm{Cs},2}$ correspond to Cs^+ associations. Proton H^+ and Cesium Cs^+ are distinguished from other ions because of surface equilibria. All mobile ions are modelled as point-like charges.

$$\equiv \mathrm{TiO}^{-\frac{4}{3}} + \mathrm{H}^+ \rightleftharpoons \equiv \mathrm{TiOH}^{-\frac{1}{3}} \quad ; \quad K_{\mathrm{H},1} = \frac{\{\equiv \mathrm{TiOH}^{-\frac{1}{3}}\}}{\{\equiv \mathrm{TiO}^{-\frac{4}{3}}\}[\mathrm{H}^+]_{\mathrm{loc},j}} \,, \tag{S1}$$

$$\equiv \mathrm{TiOH}^{-\frac{1}{3}} + \mathrm{H}^+ \rightleftharpoons \equiv \mathrm{TiOH}_2^{+\frac{2}{3}} \quad ; \quad K_{\mathrm{H},2} = \frac{\{\equiv \mathrm{TiOH}_2^{+\frac{2}{3}}\}}{\{\equiv \mathrm{TiOH}^{-\frac{1}{3}}\}[\mathrm{H}^+]_{\mathrm{loc},j}} , \tag{S2}$$

$$\equiv \mathrm{TiO}^{-\frac{4}{3}} + \mathrm{Cs}^+ \rightleftharpoons \equiv \mathrm{TiOCs}^{-\frac{1}{3}} \quad ; \quad K_{\mathrm{Cs},1} = \frac{\{\equiv \mathrm{TiOCs}^{-\frac{1}{3}}\}}{\{\equiv \mathrm{TiO}^{-\frac{4}{3}}\}[\mathrm{Cs}^+]_{\mathrm{loc},j}} , \tag{S3}$$

$$\equiv \mathrm{TiOH}^{-\frac{1}{3}} + \mathrm{Cs}^+ \rightleftharpoons \equiv \mathrm{TiOHCs}^{+\frac{2}{3}} \quad ; \quad K_{\mathrm{Cs},2} = \frac{\{\equiv \mathrm{TiOHCs}^{+\frac{2}{3}}\}}{\{\equiv \mathrm{TiOH}^{-\frac{1}{3}}\}[\mathrm{Cs}^+]_{\mathrm{loc},j}} \tag{S4}$$

where $[\mathrm{H}^+]_{\mathrm{loc},j}$ and $[\mathrm{Cs}^+]_{\mathrm{loc},j}$ are local (surface) ions concentrations. Index j depicts either the inner or the outer nanotube surface. Local concentrations are functions of $\psi(r = R_{\mathrm{inner}})$ or $\psi(r = R_{\mathrm{outer}})$[16]. The interaction of surface groups is taken into account indirectly through mean field approximation[17]. The total density of sites per unit surface Γ can be written as

$$\Gamma = \{\equiv \mathrm{TiO}^{-\frac{4}{3}}\} + \{\equiv \mathrm{TiOH}^{-\frac{1}{3}}\} + \{\equiv \mathrm{TiOH}_2^{+\frac{2}{3}}\} + \{\equiv \mathrm{TiOCs}^{-\frac{1}{3}}\} + \equiv \mathrm{TiOHCs}^{+\frac{2}{3}}\} \ . \tag{S5}$$

Each site has net charge z_x, the number of bonded (associated) Cs^+ cations $N_1^{\mathrm{Cs}^+}$, and the number of bonded H^+ cations $N_1^{\mathrm{H}^+}$. To derive the expression for the surface charge density, we can define a

parameter $f_{x,j}$ as a fraction of the surface concentration of the particular site, compared to the total site density

$$f_{x,j} = \frac{\{\equiv \text{site}\}}{\Gamma} \quad . \tag{S6}$$

where x depicts the site. By combining Eq. S1-S4, we can express sites populations for any particular bulk pH and Cs^+ concentrations as a function of local potential (local concentrations at the interface)

$$\begin{aligned}
f_{1,x} &= \frac{1}{1+K_{\mathrm{H},1}[\mathrm{H}^+]_{\mathrm{loc},j}+K_{\mathrm{H},1}K_{\mathrm{H},2}[\mathrm{H}^+]^2_{\mathrm{loc},j}+K_{\mathrm{Cs},1}\left[\mathrm{Cs}^+\right]_{\mathrm{loc},j}+K_{\mathrm{H},1}K_{\mathrm{Cs},2}[\mathrm{H}^+]_{\mathrm{loc},j}\left[\mathrm{Cs}^+\right]_{\mathrm{loc},j}}\\
f_{2,x} &= \frac{K_{\mathrm{H},1}[H^+]_{\mathrm{loc},j}}{1+K_{\mathrm{H},1}[\mathrm{H}^+]_{\mathrm{loc},j}+K_{\mathrm{H},1}K_{\mathrm{H},2}[\mathrm{H}^+]^2_{\mathrm{loc},j}+K_{\mathrm{Cs},1}\left[\mathrm{Cs}^+\right]_{\mathrm{loc},j}+K_{\mathrm{H},1}K_{\mathrm{Cs},2}[\mathrm{H}^+]_{\mathrm{loc},j}\left[\mathrm{Cs}^+\right]_{\mathrm{loc},j}}\\
f_{3,x} &= \frac{K_{\mathrm{H},1}K_{\mathrm{H},2}[\mathrm{H}^+]^2_{\mathrm{loc},j}}{1+K_{\mathrm{H},1}[\mathrm{H}^+]_{\mathrm{loc},j}+K_{\mathrm{H},1}K_{\mathrm{H},2}[\mathrm{H}^+]^2_{\mathrm{loc},j}+K_{\mathrm{Cs},1}\left[\mathrm{Cs}^+\right]_{\mathrm{loc},j}+K_{\mathrm{H},1}K_{\mathrm{Cs},2}[\mathrm{H}^+]_{\mathrm{loc},j}\left[\mathrm{Cs}^+\right]_{\mathrm{loc},j}}\\
f_{4,x} &= \frac{K_{\mathrm{Cs},1}\left[\mathrm{Cs}^+\right]_{\mathrm{loc},j}}{1+K_{\mathrm{H},1}[\mathrm{H}^+]_{\mathrm{loc},j}+K_{\mathrm{H},1}K_{\mathrm{H},2}[\mathrm{H}^+]^2_{\mathrm{loc},j}+K_{\mathrm{Cs},1}\left[\mathrm{Cs}^+\right]_{\mathrm{loc},j}+K_{\mathrm{H},1}K_{\mathrm{Cs},2}[\mathrm{H}^+]_{\mathrm{loc},j}\left[\mathrm{Cs}^+\right]_{\mathrm{loc},j}}\\
f_{5,x} &= \frac{K_{\mathrm{H},1}K_{\mathrm{Cs},2}[\mathrm{H}^+]_{\mathrm{loc},j}\left[\mathrm{Cs}^+\right]_{\mathrm{loc},j}}{1+K_{\mathrm{H},1}[\mathrm{H}^+]_{\mathrm{loc},j}+K_{\mathrm{H},1}K_{\mathrm{H},2}[\mathrm{H}^+]^2_{\mathrm{loc},j}+K_{\mathrm{Cs},1}\left[\mathrm{Cs}^+\right]_{\mathrm{loc},j}+K_{\mathrm{H},1}K_{\mathrm{Cs},2}[\mathrm{H}^+]_{\mathrm{loc},j}\left[\mathrm{Cs}^+\right]_{\mathrm{loc},j}} \quad .
\end{aligned} \tag{S7}$$

Finally, we can define the surface charge density of the exposed surface σ_j as a function of local ion concentrations or the electrostatic potential at the interface

$$\sigma_j = e\Gamma \sum_{x=1}^{5} z_x f_{x,j} . \tag{S8}$$

5.2 Electrostatics of the system through Poisson-Boltzmann calculations

To account for the electrostatics of the system composed of single, infinitely long TiO_2 NT immersed in the aqueous solution of ions, we adopted the classical Density Functional Theory of inhomogenous Coulomb fluids[18]. We considered a cell model in which infinitely long TiO_2 NT is placed. Coulomb interactions between colloid particles should diminish in distance less than d_{mean} for this approximation to hold, where d_{mean} is the mean distance between colloid particles in the uniform suspension[19]. It was shown before that the cell model operates safely within $\kappa d_{\mathrm{mean}} < 1$, which means for higher salt concentrations or lower suspension densities. In our work, cell model approximation is made even at low $CsNO_3$ concentrations and at pH around 7.

5.2.1 Validity of cell model through experimental argument

Practically, it is very difficult to experimentally determine d_{mean} between TiO_2 NTs, due to the agglomeration phenomenon and co-existence of amorphous TiO_2 which is partially retained even after the high-temperature annealing. The ideal experimental procedure of dispersing the nanomaterials in the aqueous solution would result with all nanoparticles being detached and uniformly distributed[20]. In the case of TiO_2 NTs, one cannot use long duration of high-power ultrasound probe dispersion method since it causes fragmentation and breakage of tubular structures[21–23]. Due to the fragility of the multi-layered structure, it is almost impossible to detach single nanotubes completely, which leaves an unknown fraction of TiO_2 NTs attached as agglomerates, rather than loose nanotubes in the aqueous suspension. In this study, we optimized dispersion conditions and showed that the ultrasound probe method causes the bimodal distribution of TiO_2 NTs, suggesting the moderate success of detachment of agglomerates into loose nanotubes (see Figure 3). From a practical point of view, we consider a cell model for all ranges of calculations.

5.2.2 Free energy functional

Ions are considered as an ideal gas under influence of the external electric field where the source is TiO_2 NT. Ions interact with both the inner and outer surfaces of the nanotube. Ion-ion interactions are Coulombic only. The grand canonical potential functional can be written as[24]

$$\begin{aligned}\Omega[\rho_\alpha(\mathrm{r})] = k_\mathrm{b}T\sum_\alpha\int \mathrm{d}^3r\,\rho_\alpha(\mathrm{r})\left(\ln\left(\frac{\rho_\alpha(\mathrm{r})}{\rho_\alpha^0}\right)-1\right) + \\ k_\mathrm{B}T\int\sum_\alpha z_\alpha\rho_\alpha(\mathrm{r})\Phi(\mathrm{r})\mathrm{d}^3r\end{aligned} \tag{S9}$$

where $\rho_\alpha(\mathrm{r})$, z_α are respectively one-body ion densities and the charge of the ions α. ρ_α^0 is reservoir ion concentration while $\Phi(r) = e\Psi(r)/k_\mathrm{B}T$ is the 'dimensionless' potential calculated from Coulomb law. $\Psi(r)$ is the electrostatic potential, e is the elementary charge, k_B is the Boltzmann constant and T is the thermodynamic temperature. Minimisation of grand canonical functional yields usual Boltzmann distributions:

$$\rho_\alpha(r) = \rho_\alpha^0\exp\left(-z_\alpha\Phi(r)\right) \tag{S10}$$

Inserting Eq. S10 into Poisson equation yields

$$\Delta\Phi(r) = -4\pi l_\mathrm{B}\sum_\alpha z_\alpha\rho_\alpha^0\exp\left(-z_\alpha\Phi(r)\right) \tag{S11}$$

where $l_\mathrm{B} = e^2/4\pi k_\mathrm{B}T\varepsilon_0\varepsilon_{\mathrm{r},1}$ is the Bjeruum length and ε_0 is the vacuum permittivity. $\kappa = \left(4\pi l_\mathrm{B}\sum_\alpha z_\alpha^2\rho_\alpha^0\right)^{1/2}$ is the inverse Debye length. In a cylindrical coordinate system, the radial component of the Laplace operator reads $\frac{\mathrm{d}^2}{\mathrm{d}r^2} + \frac{1}{r}\frac{\mathrm{d}}{\mathrm{d}r}$ and the Poisson-Boltzmann equation reads

$$\frac{\mathrm{d}^2\Phi(r)}{\mathrm{d}r^2} + \frac{1}{r}\frac{\mathrm{d}\Phi(r)}{\mathrm{d}r} = -4\pi l_\mathrm{B}\sum_\alpha z_\alpha\rho_\alpha^0\exp\left(-z_\alpha\Phi(r)\right). \tag{S12}$$

The boundary conditions are given by Gauss's Law[25,26]. The derivation is already published in the article by Špadina *et al.*[3]. Here we repeat the original derivation since it is needed for later model upgrades. In the centre of cylinder, the electric field goes to zero due to the symmetry. The first boundary condition for $\Phi(r = 0)$ is

$$\left.\frac{\mathrm{d}\Phi(r)}{\mathrm{d}r}\right|_{r=0} = 0\ . \tag{S13}$$

At the inner nanotube surface ($r \to R_\mathrm{inner}$) the second boundary condition states

$$\left.\frac{\mathrm{d}\Phi(r)}{\mathrm{d}r}\right|_{R_\mathrm{i}} = -\frac{e\int_0^{R_\mathrm{inner}}\rho_{\mathrm{el},1}\,r\,\mathrm{d}r}{\varepsilon_0\varepsilon_{\mathrm{r},1}k_\mathrm{B}TR_\mathrm{i}} \tag{S14}$$

where $\rho_{\mathrm{el},1}$ is the electrolyte charge density inside of cylinder. The electrolyte charge density is defined by

$$\rho_{\mathrm{el},j}(r) = e\sum_{\alpha=1}^{N} z_\alpha\rho_\alpha(r) \tag{S15}$$

where index j defines the inner or outer nanotube surface.

The $\Phi(r)$ through TiO_2 layer is obtained by integration of the Gauss Law. The following function is obtained:

$$\Phi(r) = \Phi(R_\mathrm{inner}) + \frac{e\int_0^{R_\mathrm{inner}}\rho_{\mathrm{el},1}\,r\,\mathrm{d}r}{\varepsilon_0\varepsilon_{\mathrm{r},2}k_\mathrm{B}T}\ln\left(\frac{R_\mathrm{inner}}{r}\right) + \frac{e\sigma_\mathrm{inner}R_\mathrm{inner}}{\varepsilon_0\varepsilon_{\mathrm{r},2}k_\mathrm{B}T}\ln\left(\frac{R_\mathrm{inner}}{r}\right) \tag{S16}$$

for $R_{\text{inner}} \le r \le R_{\text{outer}}$, where $\Phi(R_i)$ is the integration constant which is given from continuity of potential at the inner TiO_2 NT surface.

At the outer cylinder surface ($r \to R_{\text{outer}}$) Gauss theorem expresses the electric field as a function of the total internal charge. The latter is a sum of the volume integral of the inner cylinder electrolyte charge density and charge on the inner and outer surfaces. Consequently, the boundary condition for the potential outside the cylinder reads

$$\left.\frac{\mathrm{d}\Phi(r)}{\mathrm{d}r}\right|_{R_{\text{outer}}} = -\frac{e\int_0^{R_{\text{inner}}}\rho_{\text{el},1}\, r\, dr}{\varepsilon_0\varepsilon_{\text{r},1}k_{\text{B}}TR_{\text{outer}}} - \frac{e\sigma_{\text{inner}}R_{\text{inner}}}{\varepsilon_0\varepsilon_{\text{r},1}k_{\text{B}}TR_{\text{outer}}} - \frac{e\sigma_{\text{outer}}}{\varepsilon_0\varepsilon_{\text{r},1}k_{\text{B}}T} \tag{S17}$$

Far from the cylinder's outer surface, the potential goes to zero

$$\Phi(r >> \kappa^{-1}) = 0 \tag{S18}$$

The solution of Eq. S11 for both cases (inside and outside) with respect to boundary conditions yields the dimensionless potential which is at the end converted to the electrostatic potential $\Psi(r) = k_{\text{B}}T\,\Phi(r)/e$.

With this general framework being established, any ion can be added to the model. In the following sections, we will introduce the HCO_3^- and CO_3^{2-} ions that originate from CO_2 dissolution during the titration experiment under the air atmosphere or as contaminants in the inert gas setup. Furthermore, we will then combine all equations and express them in a manner compatible with a comparison with the actual titration experiment (Figure 5).

5.3 Correction due to dissolution of CO_2 within titration experiment

To represent the effect of CO_2 as a contaminant in acid-based titrations of nanomaterials suspension we consider the following set of chemical equilibria:

$$\begin{aligned}
&\mathrm{CO_2(g) = CO_2(aq)}\ ;K^\circ = \frac{[\mathrm{CO_2}]_{\text{aq}}}{p_{\mathrm{CO_2}}/p^\circ}\\
&\mathrm{CO_2(aq) + H_2O = HCO_3^- + H^+}\quad ;\mathrm{K_{A1}}\\
&\mathrm{HCO_3^- = CO_3^{2-} + H^+}\quad ;\mathrm{K_{A2}}
\end{aligned} \tag{S19}$$

where $\mathrm{pK_{A1}} = 6.6$, and $\mathrm{pK_{A2}} = 10.1$. The aim is to calculate the total concentration of dissolved CO_2 in the a solution or suspension $[\mathrm{CO_2}]_{\text{aq}}^{\text{tot}}$, as a function of pH.

We can express dissolved species as:

$$[\mathrm{CO_2}]_{\text{aq}} = K^\circ p_{\mathrm{CO_2}}/p^\circ \tag{S20}$$

$$\left[\mathrm{HCO_3^-}\right] = \frac{10^{-\mathrm{pK_{A1}}}[\mathrm{CO_2}]_{\text{aq}}}{10^{-\mathrm{pH}}} = \frac{10^{-\mathrm{pK_{A1}}}K^\circ p_{\mathrm{CO_2}}/p^\circ}{10^{-\mathrm{pH}}}\ , \tag{S21}$$

$$\left[\mathrm{CO_3^{2-}}\right] = \frac{10^{-\mathrm{pK_{A1}}}\left[\mathrm{HCO_3^-}\right]}{10^{-\mathrm{pH}}} = \frac{10^{-(\mathrm{pK_{A1}}+\mathrm{pK_{A2}})}K^\circ p_{\mathrm{CO_2}}/p^\circ}{10^{-2\mathrm{pH}}}\ . \tag{S22}$$

The total concentration of dissolved CO_2 can be written as $[\mathrm{CO_2}]_{\text{aq}}^{\text{tot}} = [\mathrm{CO_2}]_{\text{aq}} + \left[\mathrm{HCO_3^-}\right] + \left[\mathrm{CO_3^{2-}}\right]$. When Eq. S20, S21, and S22 are used, the following expression is obtained:

$$[\mathrm{CO_2}]_{\text{aq}}^{\text{tot}} = \left(1 + \frac{10^{-\mathrm{pK_{A1}}}}{10^{-\mathrm{pH}}} + \frac{10^{-(\mathrm{pK_{A1}}+\mathrm{pK_{A2}})}}{10^{-2\mathrm{pH}}}\right)K^\circ p_{\mathrm{CO_2}}/p^\circ \tag{S23}$$

where $K^\circ p_{\mathrm{CO_2}}/p^\circ$ factor is evaluated by using values of the equilibrium CO_2 dissolution constant ($K^\circ = 0.035$) taken from the reference[27,28]. Note that $K^\circ = 0.035$ is the value measured for the NaCl solution, which we consider similar to our $CsNO_3$ aqueous solution.

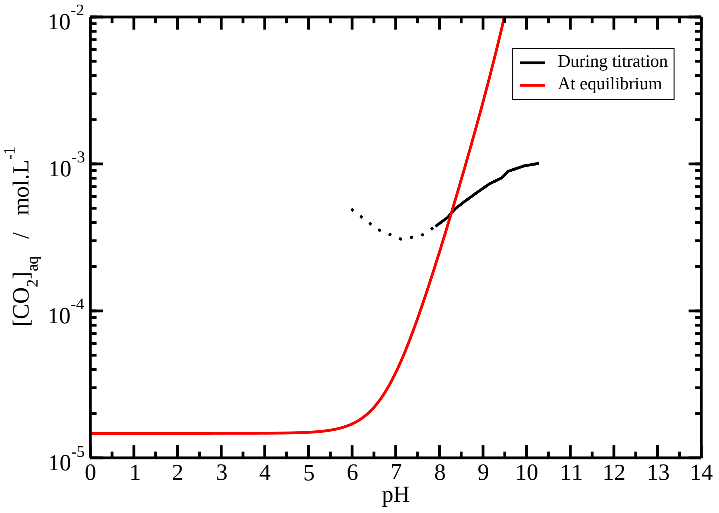

Fig. S8 The concentration of dissolved CO_2 ($[CO_2]_{aq}^{tot}$) as a function of pH. Red line shows theoretical value of $[CO_2]_{aq}^{tot}$ in the aqueous solution. Black dots show $[CO_2]_{aq}^{tot}$ values obtained by fitting the blank titration experiment.

At this point $[CO_2]_{aq}^{tot}$ is defined for any pH and salt concentration. Note that during the potentiometric titration of the suspension, pH and some of ions concentrations change. Therefore, the next step is to connect $[CO_2]_{aq}^{tot}$ and the resulting carbonate species in the aqueous solution with the volume of added titrant. This will be explained in the following section called '*Concentration of ions within the titration experiment*'. In the section called '*Fitting the blank acid-base titration to obtain* $[CO_2]_{aq}^{tot}$' the procedure to fit $[CO_2]_{aq}^{tot}$ to the experimental blank titration curve with Eq. S23.

5.4 Concentration of all ions within the titration experiment

5.4.1 Practical example: the titration with CsOH titrant that starts from the acidic medium at a given $CsNO_3$ concentration

The titration was initiated from acidic conditions, initially adjusted by standardized HNO_3. During the titration, CsOH is added in small volume while stirring V_d. To connect speciation and pH we need to express $V_d(CsOH)$. From the conservation of charge (the electroneutrality in the bulk), we have $[Cs^+]+[H^+]=[NO_3^-]+[OH^-]+[HCO_3^-]+2[CO_3^{2-}]$. Note that $[H^+]$, $[OH^-]$, $[HCO_3^-]$, $[CO_3^{2-}]$ are known from pH, while $[Cs^+]$, and $[NO_3^-]$ are known from dilution (the addition of the base within the titration experiment) but concentration needs to be systematically modified for dilution factor for any $V_d(CsOH)$. Initially for $CsNO_3$ solution with adjusted pH by the addition of nitric acid to acidic region we have:

$$[Cs^+]=\frac{c(CsNO_3)_0 V(CsNO_3)_0}{V(HNO_3)_0+V(CsNO_3)_0}\,, \tag{S24}$$

where $V(HNO_3)_0$ is the volume of the added HNO_3. Note that our particular experiments start at 25 ml deionized water V_0 in which $V(HNO_3)_0$ is added to set the initial pH. For any addition of the CsOH base within the span of the titration, we further change the total volume of the mixture, thus we have to modify Eq. S24 for the addition of the finite volume of the titrant:

$$[Cs^+]=\frac{c(CsNO_3)_0 V(CsNO_3)_0+c(CsOH)_0 V_d(CsOH)}{V(HNO_3)_0+V(CsNO_3)_0+V_d(CsOH)}\,. \tag{S25}$$

while for the nitrate anion, the dilution yields

$$\left[NO_3^-\right] = \frac{c(HNO_3)_0 V(HNO_3)_0 + c(CsNO_3)_0 V(CsNO_3)_0}{V(HNO_3)_0 + V(CsNO_3)_0 + V_d(CsOH)} \ . \tag{S26}$$

Carbonate species that originate by dissolution of CO_2 (see Eq. S23):

$$\left[H^+\right] = 10^{-pH} \ , \tag{S27}$$

$$\left[OH^-\right] = 10^{-14+pH} \ , \tag{S28}$$

$$\left[HCO_3^-\right] = \frac{10^{-pK_{A1}+pH}}{1+10^{-pK_{A1}+pH}+10^{-pK_{A1}-pK_{A2}+2pH}} \times [CO_2]_{aq}^{tot} \ , \tag{S29}$$

$$\left[CO_3^{2-}\right] = \frac{10^{-pK_{A1}-pK_{A2}+2pH}}{1+10^{-pK_{A1}+pH}+10^{-pK_{A1}-pK_{A2}+2pH}} \times [CO_2]_{aq}^{tot} \ . \tag{S30}$$

Now that we have expressions for all ions in the system, we can use the electroneutrality condition to declare

$$\left[Cs^+\right] - \left[NO_3^-\right] = A \tag{S31}$$

and substitute expressions Eq. S25 S26 into Eq. S31. After some rearrangement we isolate V_B

$$V_d(CsOH) = \frac{A(V(HNO_3)_0 + V(CsNO_3)_0 + V_d(CsOH)) + c(HNO_3)_0 V(HNO_3)_0}{c(CsOH)_0} \tag{S32}$$

with $A = \left[OH^-\right] - [H^+] + \left[HCO_3^-\right] + 2\left[CO_3^{2-}\right]$. Finally Eq. S27-S30 can be introduced to the denominator of Eq. S32 and we obtain the full expression for the volume of added CsOH base needed to obtain certain pH when CO_2 is dissolved during the titration experiment. Note that we still need a method to calculate or obtain $[CO_2]_{aq}^{tot}$. This is described in the following section.

5.5 Fitting the blank acid-base titration to obtain $[CO_2]_{aq}^{tot}$

To obtain $[CO_2]_{aq}^{tot}$ we exploit the blank titration at the given salt concentration.

The concentrations of both $\left[HCO_3^-\right]$ and $\left[CO_3^{2-}\right]$ ions are fitted on the total solubility of CO_2. This total solubility includes the three CO_2 species. We use for this total solubility an analytical formula:

$$[CO_2]_{aq}^{tot} = a_2(\mathrm{arctg}(a_1(pH - a_0)) + \pi/2) \tag{S33}$$

with $a_2 = 4.31189 10^4$, $a_1 = 0.7814448$ and $a_0 = 8.719$. This is the continuous blue line on Figure 9. Once the good fit is obtained, Eq. S28, S29, S30 and give speciation of OH^-, CO_3^{2-}, and HCO_3^- for any given pH(V_B).

5.5.1 Obtaining CO_3^{2-}, and HCO_3^- speciation in the TiO_2 NTs suspension

Experimentally, pH vs V_B data were collected in same time intervals. Now we make an approximation that the dissolution rate of CO_2 is the same in blank titration and the TiO_2 NTs suspension titration. Therefore, our approximation allows to equalize ions speciation (Eq. Eq. S28, S29, S30) in both blank and TiO_2 NTs suspension titrations. While in realistic case, there is the effect of the nanomaterial onto dissolution rate and subsequent equilibria, it is less pronounced than the dominant pH effect.

So by fitting the blank titration and using the aforementioned expressions, we have the full speciation of all ions during the TiO_2 NTs titration experiment. It must be noted that by fitting we have obtain total average concentrations of ions. These quantities need to be imported in Poisson-Boltzmann calculations as total average concentrations, rather than the bulk (the reservoir) concentrations.

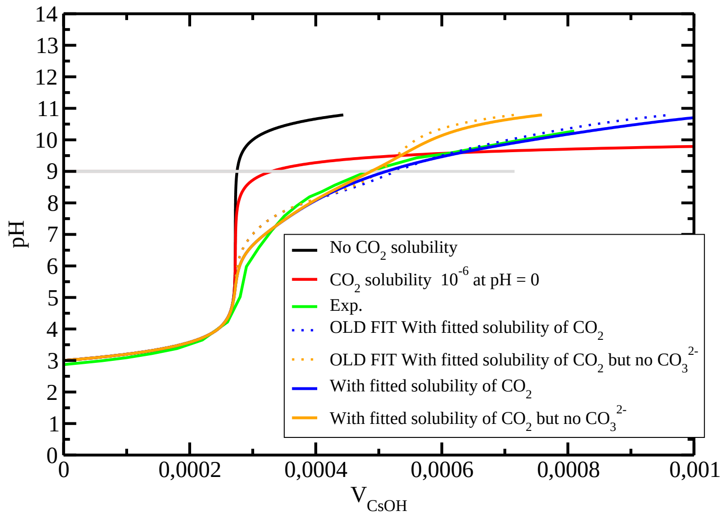

Fig. S9 Fitting procedure with arcus tangens to deduce $[CO_2]_{aq}^{tot}$ from the blank titration at the given total salt concentration. pH or the suspension as a function of added base (CsOH). Green line depicts experimental, while other curves depict different levels of approximation to achieve the best fit. From the best fit, the full speciation versus pH is calculated vie Eq. S27-S30 and imported into PB model.

5.6 Calculation of Cs^+ distributions from P.B. in the aqueous suspension of TiO_2 NTs

Mass density

$$\gamma = \frac{kg}{m^{-3}} \tag{S34}$$

Specific surface

$$s = \frac{m^2}{kg} \tag{S35}$$

We define: surface per length

$$S_\mathrm{L} = \frac{2\pi R_\mathrm{inner} L + 2\pi R_\mathrm{outer} L}{L} = 2\pi \left(R_\mathrm{inner} + R_\mathrm{outer}\right) \tag{S36}$$

and length of nanotubes per volume of solution

$$\Lambda_\mathrm{V} = \frac{\gamma s}{w} = \frac{\gamma s}{2\pi \left(R_\mathrm{inner} + R_\mathrm{outer}\right)}, \tag{S37}$$

because γs is the total surface per unit volume (liter).

By the unit length of nanotube, we have:

$$\left[\mathrm{Cs}^+\right]_\mathrm{l}^\mathrm{total} = \left[\mathrm{Cs}^+\right]_\mathrm{l}^\mathrm{res} + \left[\mathrm{Cs}^+\right]_\mathrm{l}^\mathrm{excess} \tag{S38}$$

where $[\mathrm{Cs}^+]_\mathrm{l}^\mathrm{excess}$ is the difference due to nanotube. For a given length L of nanotube we define

$$\left[\mathrm{Cs}^+\right]_\mathrm{l} = \frac{n(\mathrm{Cs}^+)}{length\ of\ nanotube} \tag{S39}$$

therefore the distribution of Cs^+ ions per length of nanotube is

$$[\mathrm{Cs}^+]_\mathrm{l} = \int_0^{+\infty} [\mathrm{Cs}^+]^\mathrm{res} e^{-\beta e \psi} 2\pi r \mathrm{d}r \tag{S40}$$

where β is Boltzmann factor $\beta = 1/k_\mathrm{B}T$, ψ is electrostatic potential, r is distance from the center of coordinate system. It is important to stress that in solid part TiO_2NTs between the inner and outer surface $R_\mathrm{inner} < r < R_\mathrm{outer}$ there are no ions (impermeable wall) so the integral in Eq. S40 needs to be split into interior and exterior of nanotubes. Note that $[\mathrm{Cs}^+]^\mathrm{res}$ from equation above has units of volume, while $[\mathrm{Cs}^+]_\mathrm{l}$ is in unit length. Now after rearrangement of Eq. S38 and the inclusion of Eq. S40 we obtain

$$[\mathrm{Cs}^+]_\mathrm{l}^\mathrm{excess} = \int_0^{+\infty} [\mathrm{Cs}^+]^\mathrm{res} \left(e^{-\beta e \psi} - 1\right) 2\pi r \mathrm{d}r + 2\pi \left(R_\mathrm{inner}\sigma_\mathrm{inner}^{\mathrm{Cs}^+} + R_\mathrm{outer}\sigma_\mathrm{outer}^{\mathrm{Cs}^+}\right) \tag{S41}$$

where $\sigma_\mathrm{inner}^{\mathrm{Cs}^+}$ and $\sigma_\mathrm{outer}^{\mathrm{Cs}^+}$ are concentrations of Cs^+ per unit of surface for inner and outer surface of nanotube. In program, $\sigma_\mathrm{inner}^{\mathrm{Cs}^+}$ and $\sigma_\mathrm{outer}^{\mathrm{Cs}^+}$ are calculated (sampled) using generalized equation for surface charge density

$$\sigma_j^{\mathrm{Cs}^+} = \Gamma \sum_{x=1}^{5} N_x^{\mathrm{Cs}^+} f_{x,j} \tag{S42}$$

where Γ is the site density per unit surface, index j defines inner or outer surface, $N_\mathrm{l}^{\mathrm{Cs}^+}$ is the number of bonded (associated) Cs^+ that occupy particular sites x. We distinguish 5 different sites, two of which have bonded Cs^+. All sites, their charges and number of associated ions are presented in detail further in the text. $\sigma_\mathrm{inner}^{\mathrm{Cs}^+}$ and $\sigma_\mathrm{outer}^{\mathrm{Cs}^+}$ are functions of electrostatic potentials at the two solid/electrolyte solution interfaces. If we combine it with Eq. S37 we obtain the total Cs^+ concentration per unit volume is then

$$[\mathrm{Cs}^+]^\mathrm{total} = [\mathrm{Cs}^+]_\mathrm{l}^\mathrm{excess}\Lambda_\mathrm{V} + [\mathrm{Cs}^+]^\mathrm{res} \tag{S43}$$

so finally we get

$$[\mathrm{Cs}^+]^\mathrm{total} = \frac{\gamma s}{R_\mathrm{inner} + R_\mathrm{outer}} \left[\int_0^{+\infty} [\mathrm{Cs}^+]^\mathrm{res} \left(e^{-\beta e \psi} - 1\right) r \mathrm{d}r + R_\mathrm{inner}\sigma_\mathrm{inner}^{\mathrm{Cs}^+} + R_\mathrm{outer}\sigma_\mathrm{outer}^{\mathrm{Cs}^+}\right] + [\mathrm{Cs}^+]^\mathrm{res} \tag{S44}$$

and total nitrate total concentration

$$[\mathrm{NO}_3^-]^\mathrm{total} = \frac{\gamma s}{R_\mathrm{inner} + R_\mathrm{outer}} \left[\int_0^{+\infty} [\mathrm{NO}_3^-]^\mathrm{res} \left(e^{\beta e \psi} - 1\right) r \mathrm{d}r\right] + [\mathrm{NO}_3^-]^\mathrm{res} \tag{S45}$$

5.7 General relation between V_B and $[\mathrm{Cs}^+]^\mathrm{total}$, *i.e.*, how to connect the model and the experiments

We need to calculate $V_\mathrm{d}(\mathrm{CsOH})$ as a function of pH and compare it with experimental pH *vs* $V_\mathrm{d}(\mathrm{CsOH})$. Within the addition of the base from acidic to basic region, volumes of CsOH are added and the total concentration of the Cs^+ is modified (accumulated) according to following relation:

$$[\mathrm{Cs}^+]^\mathrm{total} = \frac{n(\mathrm{Cs}^+)^\mathrm{total}}{V_\mathrm{total}} = \frac{c(\mathrm{CsNO_3})_0 V(\mathrm{CsNO_3})_0 + c(\mathrm{CsOH})_0 V_\mathrm{d}(\mathrm{CsOH})}{V(\mathrm{HNO_3})_0 + V(\mathrm{CsNO_3})_0 + V_\mathrm{d}(\mathrm{CsOH})} \ . \tag{S46}$$

while for the nitrate anion, we have:

$$[\mathrm{NO}_3^-]^\mathrm{total} = \frac{c(\mathrm{HNO_3})_0 V(\mathrm{HNO_3})_0 + c(\mathrm{CsNO_3})_0 V(\mathrm{CsNO_3})_0}{V(\mathrm{HNO_3})_0 + V(\mathrm{CsNO_3})_0 + V_\mathrm{d}(\mathrm{CsOH})} \ . \tag{S47}$$

After some rearrangement we get:

$$V_\mathrm{d}(\mathrm{CsOH}) = \frac{[\mathrm{Cs}^+]^\mathrm{total}(V(\mathrm{HNO_3})_0 + V(\mathrm{CsNO_3})_0) - c(\mathrm{CsNO_3})_0 V(\mathrm{CsNO_3})_0}{c(\mathrm{CsOH})_0 - [\mathrm{Cs}^+]^\mathrm{total}} \tag{S48}$$

Now to connect the ion distribution obtained from PB model with the analytical chemistry we identify Eq. S44 with Eq. S46 and we identify Eq. S45 with Eq. S47. In this way we can obtain 'the spatial distribution' of all ions during the span of the titration experiment.

5.8 Numerical procedure: implementing the model

The goal of the simulations is to produce numerically what the experiment produces, *i.e.* pH *versus* V_B. These two data are the genuine parameters of the experiment, since, along the titration, V_B is the only driving quantity and the pH is the only measured value.

The calculations are based on Poisson Boltzmann (PB) equations for the liquid part (aqueous solution) of the system (Eq. S12), on adsorption constants for the surface terms (Eq. S7 and S8), on Gauss law inside the TiO_2 wall (Eq. S16), and again on PB outside the tube. The electric potential is continuous, as required, and its derivative is not, due to Dirac distribution of surface charges (Eq.S17). There are 5 adjustable parameters in the 1-site model calculation: the surface density of adsorption sites γ, and 4 parameters which express the association of Cs^+ and H^+ (Eq. S1 - S4). In the aqueous solution, ion distribution functions of each solvated species are the product of the reservoir (Long Distance from the tube, LD) by the electric Boltzmann term (*e.g.* for Cs^+ Eq. S10). LD (the reservoir or the bulk) is chosen as 6 Debye lengths in our simulations. As so, it is a varying parameter, depending on the concentrations of all species, *i.e.,* during the titration LD changes.

5.8.1 Program flowchart

The program flowchart includes two nested loops: the inner loop on electric potential, the outer loop on concentrations.

- input V_B, the added volume of CsOH. This gives the Total Concentrations (TC) of Cs^+ and NO_3^-, from the experiment ($[Cs^+]_{exp}^{total}$ and $\left[NO_3^-\right]_{exp}^{total}$).

- guess is made for the reservoir Cs^+ and NO_3^- ions concentrations $[Cs^+]^{res}$ and $\left[NO_3^-\right]^{res}$. The electric potential is close 0 at LD. These concentrations are not equal to the total average concentrations, due to adsorption and partitioning in the electric double layer. Still, they equal to them for the first guess. After each iteration in nested loops, new values of the reservoir Cs^+ and NO_3^- are obtained.

- The inclusion of the reservoir HCO_3^- and CO_3^{2-} ions concentrations $\left[HCO_3^-\right]^{res}$ and $\left[CO_3^{2-}\right]^{res}$ (Eq. S29 and S30). These are the consequence of the fitting of the blank titration curve. $\left[HCO_3^-\right]^{res}$ and $\left[CO_3^{2-}\right]^{res}$ ions depend on pH(V_B). As a result pH can only be made available when all charged species are determined, as detailed next, we use, inside the inner loop on the electric potential, the previous iteration pH for the $\left[HCO_3^-\right]^{res}$ and $\left[CO_3^{2-}\right]^{res}$ ions. This is a valid choice, since pH tends to a limiting value as electric potential iterations progress.

- electroneutrality is set at LD. Electroneutrality is based on adjustment of pH. This is because all other charged species have already been determined (Cs^+, NO_3, HCO_3^-, CO_3^{2-}), so that protons and hydroxides are the only remaining charged species. These two terms are linked by the ionic product of water. As so, each electric potential iteration output its own pH. When the inner loop converges, and the outer loop converges too, the last pH value of the inner loop is the final pH.

- iteration on potential in the center. This is a shooting process: a guess for the potential in the center of the nanotube ($r = 0$) results in a certain potential at LD. This potential should be close to zero (0) at LD. This process is based on the fact that the potential at any distance depends only on charges within this distance. The unique value of the potential is close to 0 at LD. The shooting process is the following:

  - guess of the electric potential in the center of the tube

- propagation of the potential up to the tube inner wall
- calculation of charges on the inner wall surface with the association parameters
- crossing the wall with the Gaussian law
- calculation of surface charges on the outter wall surface
- calculation of potential up to LD (6 Debye lengths)
- this potential should be very close to zero. We aim at 10^{-6} Volt.
- if the criterium is not met, we make another guess for the potential in the center changing the previous value in dichotomy-like fashion.

• summing the concentrations of all Cs^+ and NO_3^- species, including mobile ions in the electric double layer, adsorbed species onto nanotubes surface and in the reservoir (based on Eq. S44 and S45) to obtain the total average concentrations ($[Cs^+]_{\text{theory}}^{\text{total}}$ and $\left[NO_3^-\right]_{\text{theory}}^{\text{total}}$). These values are compared to known experimental ($[Cs^+]_{\text{exp}}^{\text{total}}$ and $\left[NO_3^-\right]_{\text{exp}}^{\text{total}}$). Numerically, the convergence limit is set as the absolute difference between calculated and experimental values. The absolute difference should be less than 10^{-8}mol dm^{-3}.

• making new guesses on Cs^+ and NO_3^-, based on all previous tries

The resulting system of non-linear equations has been solved with Mathematica 12., using the Numerical Differential Equation Solver with *NDSolve* command.

6 Model predictions

6.1 Fitting of the model

6.2 Full speciation of all charged species in the system for $c(\mathrm{CsNO_3}) = 0.01$ mol dm^{-3}

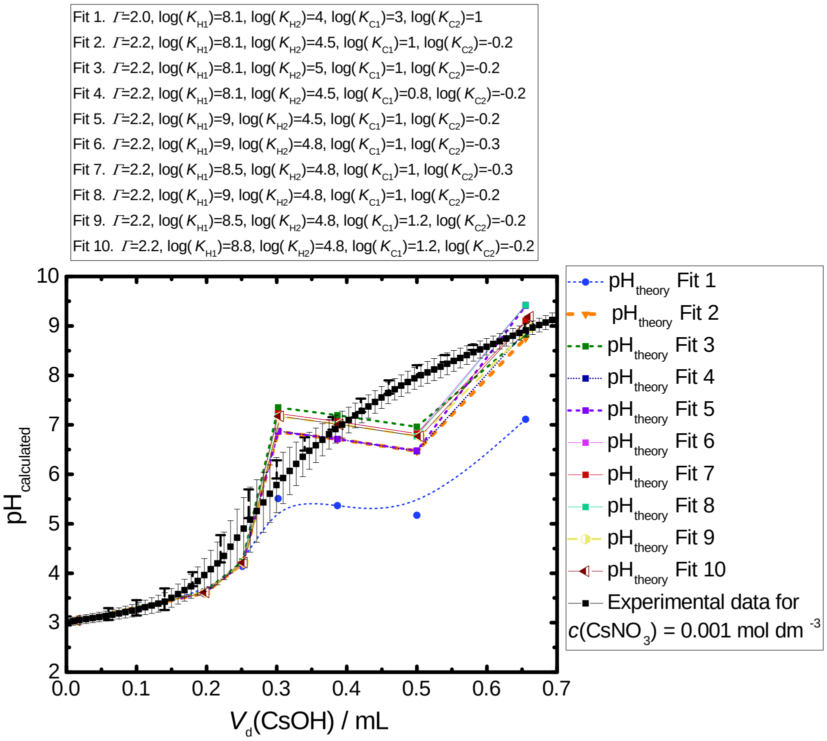

Fig. S10 Fitting - 10 best fits: comparison of calculated and experimental pH as a function of the volume of added V_d(CsOH), for $R_{inner} = 3.5$ nm, $R_{outer} = 4$ nm. Black squares depict experimental data at $c(CsNO_3) = 0.001$ mol dm^{-3}.

6.3 Surface TiO_2 NTs properties and speciation for larger radii: $R_{inner} = 4$ nm, $R_{outer} = 6$ nm

6.3.1 Dissimilarities between the inner and outer surfaces charging and cation adsorption

The differences in charging and adsorption between the two exposed surfaces of TiO_2 NTs can be expressed as the ratio of in total charge on the outer surface Q^{tot}_{outer} and the inner surface Q^{tot}_{inner}. After canceling identical factors in the expression $\frac{Q^{tot}_{outer}}{Q^{tot}_{inner}} = \frac{2L\pi R_{outer}\sigma_{outer}}{2L\pi R_{inner}\sigma_{inner}}$, the ratio is equal to $\frac{R_{outer}\sigma_{outer}}{R_{inner}\sigma_{inner}}$.

The results are plotted in Figure 14.

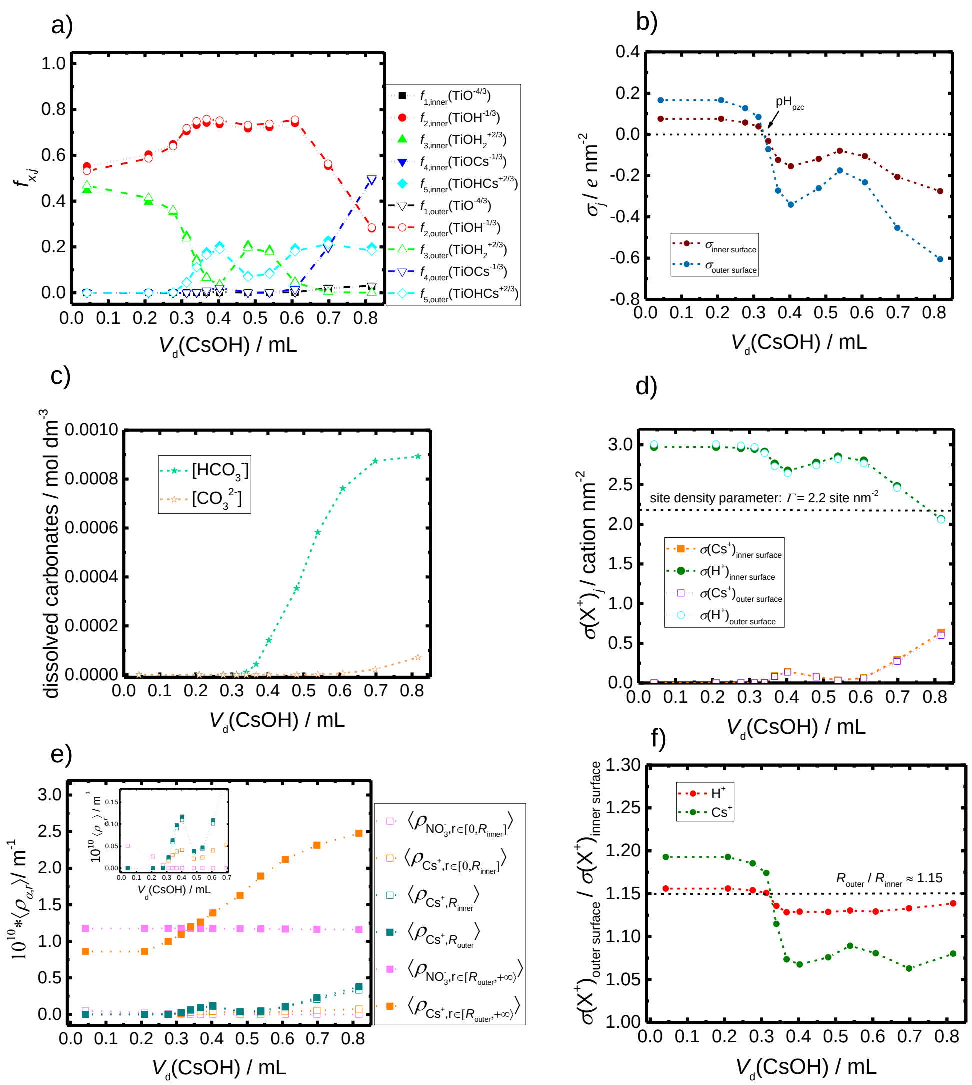

Fig. S11 Calculated surface speciation evolution within the $c(\mathrm{CsNO_3}) = 0.01$ mol dm^{-3} titration experiment. Figure shows: a) sites populations, b) surface charge densities, c) carbonate species in aqueous solution, d) surface cation coverage of surface active Cs^+ and H^+ cations, e) volume integrals of Cs^+ and NO_3^- particle distribution functions, normalized per unit length as a function of the volume of the added base in the actual titration experiment, and f) ratio of surface cation coverage on both inner and outer interface, as a function of the volume of added base in the actual titration experiment. Calculations were performed for radii $R_{\mathrm{inner}} = 3.5$ nm, $R_{\mathrm{outer}} = 4$ nm, association constants $\log K_{\mathrm{H},1} = 8.5$, $\log K_{\mathrm{H},2} = 4.8$, $\log K_{\mathrm{Cs},1} = 1.2$, and $\log K_{\mathrm{Cs},2} = -0.2$, and surface sites density $\Gamma = 2.2$

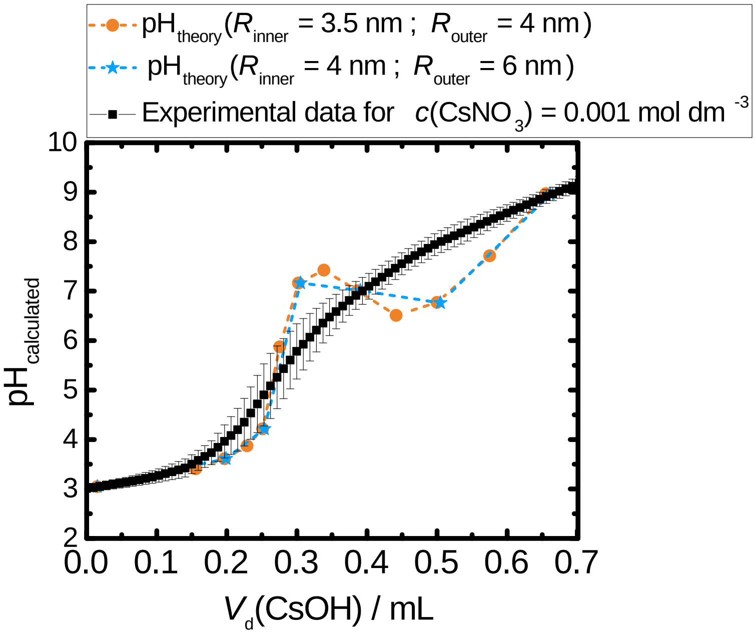

Fig. S12 Influence of radii sets used for calculation with the best fit of association constants. Calculated and average values of experimental pH are plotted as a function of the volume of added V_d(CsOH). Black squares depict experimental data at $c(CsNO_3) = 0.001$ mol dm^{-3}. Orange circles depict model predictions $R_{inner} = 3.5$ nm, $R_{outer} = 4$ nm, while blue stars depict $R_{inner} = 4$ nm, $R_{outer} = 6$ nm radii sets. Surface sites density is set to $\Gamma = 2.2$

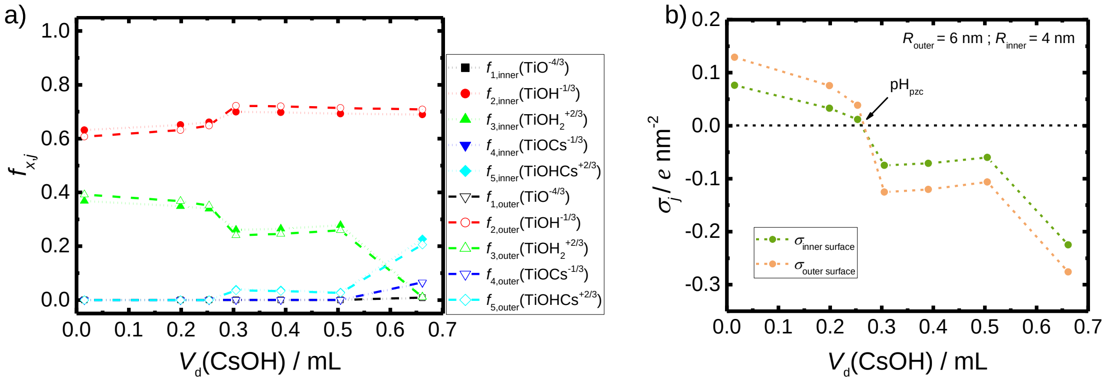

Fig. S13 Influence of radii on charge properties at $c(CsNO_3) = 0.001$ mol dm^{-3}: a) Calculated inner and outer surface charge density. b) Calculated site populations at the inner and the outer surface of TiO_2 NTs. Calculations were performed for $R_{inner} = 4$ nm, $R_{outer} = 6$ nm, , and association constants $\log K_{H,1} = 8.5$, $\log K_{H,2} = 4.8$, $\log K_{Cs,1} = 1.2$, and $\log K_{Cs,2} = -0.2$. Surface sites density is set to $\Gamma = 2.2$

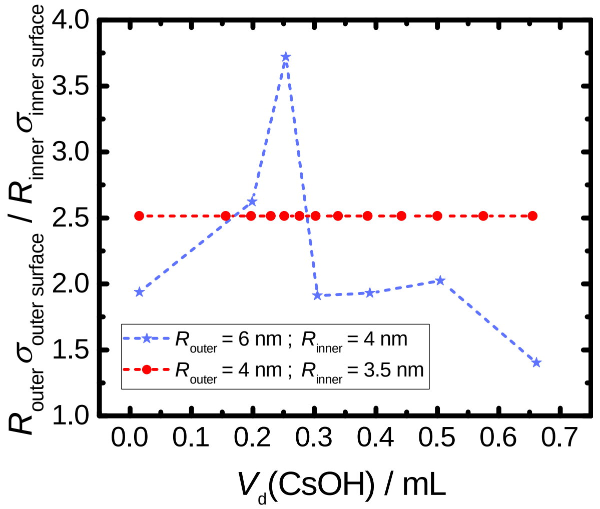

Fig. S14 Ratio of the total charge between the outer and the inner surface as a function of the added CsOH. The results are given for two different radii sets. Red circles depict $R_{\text{inner}} = 3.5$ nm, $R_{\text{outer}} = 4$ nm, while blue stars depict $R_{\text{inner}} = 4$ nm, $R_{\text{outer}} = 6$ nm radii sets. c($CsNO_3$) = 0.001 mol dm^{-3}. Surface sites density is set to $\Gamma = 2.2$

7 First-principles Molecular Dynamics Simulations

7.1 Computational Details

All the calculations were conducted using the Vienna Ab initio Simulation Package.[29] The semi-local exchange-correlation PBE functional in the generalized gradient approximation of Perdew and co-workers was used[30] along with the D2 method of Grimme to take into account the dispersion forces.[31] The electron-ion interactions were described using the projector augmented wave method[32,33] with a plane-wave cutoff kinetic energy of 400 eV. The following valence electrons were considered: $1s^1$ for H, $2s^2$ $2p^4$ for O, $3s^2$ $3p^6$ $4s^1$ $3d^3$ for Ti, $2s^2$ $2p^3$ for N, and $5s^2$ $5p^6$ $6s^1$. All the calculations were performed using the Γ-point only, due to the large size of the cell and the significant disorder of atoms. The Kohn-Sham equations[34,35] were solved self-consistently[36] until the difference in energy between cycles was lower than 10^{-4} eV. For the relaxation of the first primitive cell and of the slab prior to the DFT-MD simulations, the calculations were conducted using a 4x4x4 *k*-points grid with a kinetic cutoff energy of 1,000 eV, an energy threshold of 10^{-8} eV, and the relaxation was stopped when all forces were lower than 10-3 $eV{\cdot}A^{-1}$. Since the semi-local DFT exchange-correlation functionals such as PBE are known to inaccurately describe the high-correlated nature of Ti 3d electrons, we used the DFT+U formalism of Dudarev,[37] as implemented in VASP. The on-site Coulomb repulsion of the Ti 3d electrons was described using $U = 3.50$ eV (and $J = 0.00$ eV), considering most values used in the literature.[38–41] The *NVT* DFT-MD simulations were conducted using a Nosé-Hoover thermostat[42–44] at a temperature of 300 K with a timestep of 1 fs, allowed by replacing the mass of the proton by that of the tritium isotope. Each DFT-MD simulation was conducted during 80 ps and a thermalization period of 10 ps was excluded at the beginning of the simulation.

7.2 Structural Model

A primitive cell of anatase was generated following the experimental cell parameters $a = b = 3.7842$ Å, $c = 9.5146$ Å, and $\alpha = \beta = \gamma = 90°$ given by Horn and co-workers.[45] This quadratic cell was relaxed in terms of ion positions, cell shape, and cell size and the fully-relaxed primitive cell exhibited $a = b = 3.9344$ Å, $c = 9.7244$ Å, and $\alpha = \beta = \gamma = 90°$, which was in good accordance with the experimental parameters. Based on this relaxed primitive cell, we generated a supercell and created the (101) surface, which is known to be the most exposed surface of anatase[38,46–48]. The obtained slab was composed of three layers of 12 titanium atoms, which therefore represented a total number of 36 titanium atoms and 72 oxygen atoms (Fig. 15a). On the (101) surface, among the 12 titanium atoms that constituted the surface plane, six were five-coordinated and located slightly above the average surface plane while the six others were six-coordinated, like in the TiO_2 bulk, and located slightly below the average surface plane. Besides, the in-plane surface oxygen atoms were three-coordinated, like in the TiO_2 bulk, while six oxygen atoms were significantly above the average plane and were only two-coordinated. The ions positions of this slab was relaxed by a static DFT calculation that consisted in a series of wave function optimisation. To avoid any unwanted interaction between the two reciprocal (which existence is due to the periodic boundary conditions), a vacuum of 20 Å was added above the uppermost atom of the TiO_2 slab along the *z*-axis. Then, explicit water molecules were randomly added using the packmol software,[49] to completely fill in the aforementioned vacuum by meeting a density of 1 $g{\cdot}cm^{-3}$ for the added liquid phase. The DFT-based molecular dynamics simulations were conducted on this system. For the simulations performed with $CsNO_3$, this latter was added close to the surface by substituting four water molecules. All the molecular structures were visualized using VESTA software.[50]

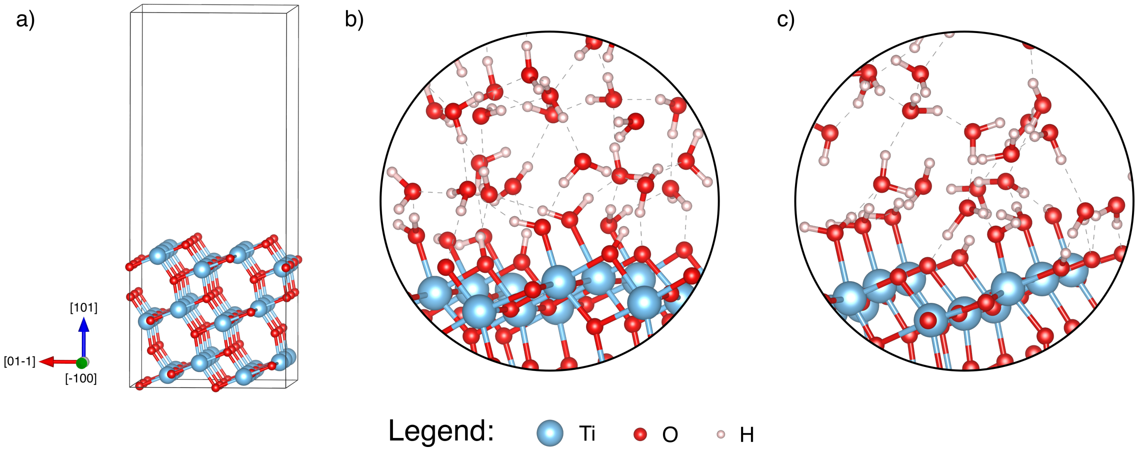

Fig. S15 a) The TiO_2 slab exposing the (101) surface used for the DFT-MD simulations seen from side. The axes represent the Miller crystallographic indices. b) Snapshot of the DFT-MD simulation of the (101) TiO_2 surface with the vacuum completely filled with water molecules, in which the surface was completely manually hydroxylated at the beginning. c) Snapshot of the DFT-MD simulation of the (101) TiO_2 surface with the vacuum completely filled with water molecules, in which all water molecules were introduced in their molecular form.

7.3 Results

7.3.1 Hydration of the (101) surface of TiO_2

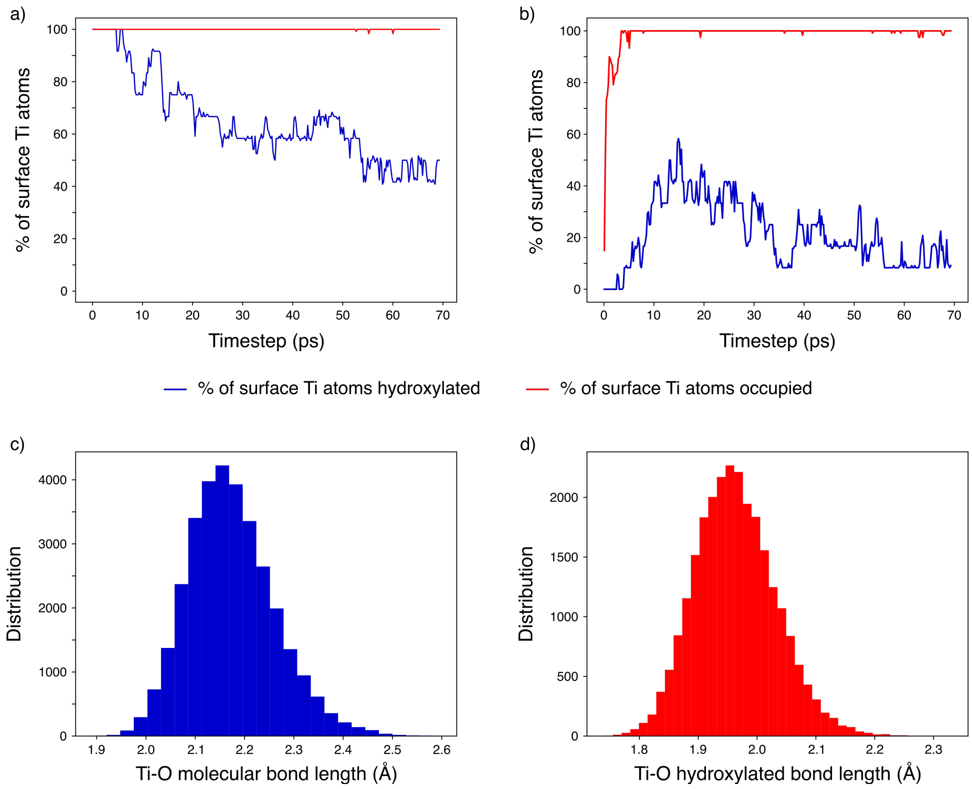

Fig. S16 a) Histogram of the Ti-O bond lengths for the surface Ti atoms that are occupied by a non-dissociated water molecule. b) Histogram of the Ti-O bond lengths for the surface Ti atoms that are occupied by a dissociated water molecule, *i.e.*, by a hydroxy group. c) Percentage of the total surface Ti atoms that are occupied either by an hydroxy group or by a non-dissociated water molecule and that are occupied by a hydroxy group as a function of time during the simulation where all the water molecules were manually dissociated in the input configuration. d) Percentage of the total surface Ti atoms that are occupied either by an hydroxy group or by a non-dissociated water molecule and that are occupied by a hydroxy group as a function of time during the simulation where all the water molecules were non-dissociated in the input configuration.

7.3.2 Adsorption of $CsNO_3$ on the (101) surface of TiO_2

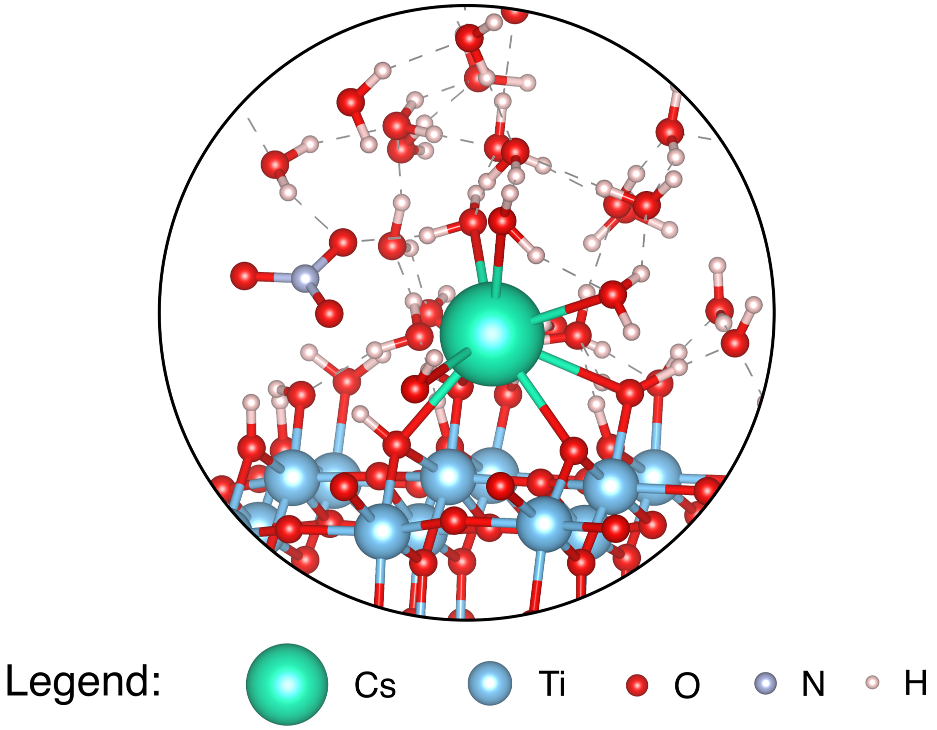

Fig. S17 Snapshot of the simulation in which the nitrate anion was initially placed at the vicinity of the TiO_2 surface along with the cesium cation, showing the diffusion of the nitrate anion to the water bulk.

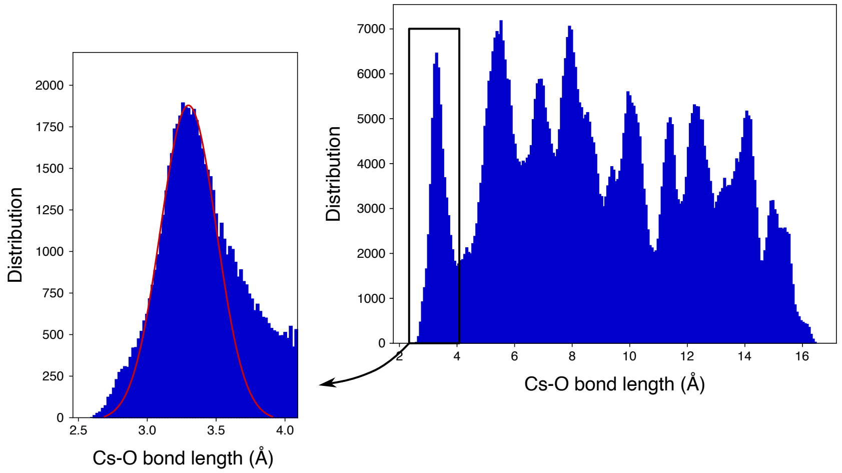

Fig. S18 Histograms of the Cs-O bond lengths on the simulation with a zoom on the first peak corresponding to the first coordination sphere.

References

[1] T. Kasuga, M. Hiramatsu, A. Hoson and T. Sekino, *Langmuir*, 1998, **7463**, 3160–3163.

[2] A. Selmani, M. Špadina, M. Plodinec, I. Delač Marion, M. G. Willinger, J. Lützenkirchen, H. D. Gafney and E. Redel, *The Journal of Physical Chemistry C*, 2015, **119**, 19729–19742.

[3] M. Špadina, S. Gourdin-Bertin, G. Dražić, A. Selmani, J.-F. Dufrêche and K. Bohinc, *ACS Applied Materials & Interfaces*, 2018, **10**, 13130–13142.

[4] D. Kowalski, D. Kim and P. Schmuki, *TiO2 nanotubes, nanochannels and mesosponge: Self-organized formation and applications*, 2013.

[5] D. Bavykin and F. Walsh, *RSC Nanoscience and Nanotechnology*, 2009, 1–182.

[6] H. Lu, J. Zhao, L. Li, J. Zheng, L. Zhang, L. Gong, Z. Wang and Z. Zhu, *Chemical Physics Letters*, 2011, **508**, 258 – 264.

[7] A. Gajović, I. Friščić, M. Plodinec and D. Iveković, *Journal of Molecular Structure*, 2009, **924-926**, 183 – 191.

[8] L. Qian, Z.-L. Du, S.-Y. Yang and Z.-S. Jin, *Journal of Molecular Structure*, 2005, **749**, 103 – 107.

[9] K. Zhang, Z. Li, S. Qi, W. Chen, J. Xie, H. Wu, H. Zhao, D. Li and S. Wang, *Chemosphere*, 2022, **290**, 132636.

[10] R. Podgornik, *The Journal of Chemical Physics*, 2018, **149**, 104701.

[11] P. Roy, S. Berger and P. Schmuki, *Angew. Chemie - Int. Ed.*, 2011, **50**, 2904–2939.

[12] D. García, J. Lützenkirchen, M. Huguenel, L. Calmels, V. Petrov, N. Finck and D. Schild, *Minerals*, 2021, **11**, 1093.

[13] G. D. Panagiotou, T. Petsi, K. Bourikas, C. S. Garoufalis, A. Tsevis, N. Spanos, C. Kordulis and A. Lycourghiotis, *Adv. Colloid Interface Sci.*, 2008, **142**, 20–42.

[14] T. Hiemstra and W. H. Van Riemsdijk, *J. Colloid Interface Sci.*, 1996, **508**, 488–508.

[15] L. Pauling, *J. Am. Chem. Soc.*, 1929, **51**, 1010–1026.

[16] K. Bourikas, T. Hiemstra and W. H. Van Riemsdijk, *Langmuir*, 2001, **17**, 749–756.

[17] N. c. v. Adžić and R. Podgornik, *Phys. Rev. E*, 2015, **91**, 022715.

[18] E. Trizac and J.-P. Hansen, *Phys. Rev. E*, 1997, **56**, 3137.

[19] Y. Hallez, J. Diatta and M. Meireles, *Langmuir*, 2014, **30**, 6721–6729.

[20] N. Mandzy, E. Grulke and T. Druffel, *Powder Technol.*, 2005, **160**, 121–126.

[21] D. Bavykin, J. Friedrich and F. Walsh, *Advanced Materials*, 2006, **18**, 2807–2824.

[22] P. O'Brien, H. Craighead and H. Kroto, *Titanate and Titania Nanotubes*, The Royal Society of Chemistry, 2009, pp. P001–P154.

[23] A. Selmani, M. Špadina, M. Plodinec, I. Delač Marion, M. G. Willinger, J. Lützenkirchen, H. D. Gafney and E. Redel, *J. Phys. Chem. C*, 2016, **120**, 4150.

[24] J.-P. Hansen and H. Löwen, *Annu. Rev. Phys. Chem.*, 2000, **51**, 209–242.

[25] K. Bohinc, J. Gimsa, V. Kralj-Iglič, T. Slivnik and A. Iglič, *Bioelectrochemistry*, 2005, **67**, 91–99.

[26] G. Allaire, J.-F. Dufrêche, A. Mikelić and A. Piatnitski, *Nonlinearity*, 2013, **26**, 881–910.

[27] S. Portier and C. Rochelle, *Chemical Geology*, 2005, **217**, 187–199.

[28] S. V. Golubev, O. S. Pokrovsky and J. Schott, *Chemical Geology*, 2005, **217**, 227–238.

[29] G. Kresse and J. Hafner, *Physical review B*, 1993, **47**, 558.

[30] J. P. Perdew, K. Burke and M. Ernzerhof, *Physical review letters*, 1996, **77**, 3865.

[31] S. Grimme, *Journal of computational chemistry*, 2006, **27**, 1787–1799.

[32] P. E. Blöchl, *Physical review B*, 1994, **50**, 17953.

[33] G. Kresse and D. Joubert, *Physical review b*, 1999, **59**, 1758.

[34] P. Hohenberg and W. Kohn, *Phys. Rev.*, 1964, **136**, B864–B871.

[35] W. Kohn and L. J. Sham, *Phys. Rev.*, 1965, **140**, A1133–A1138.

[36] G. Kresse and J. Furthmüller, *Physical review B*, 1996, **54**, 11169.

[37] S. L. Dudarev, G. A. Botton, S. Y. Savrasov, C. J. Humphreys and A. P. Sutton, *Phys. Rev. B*, 1998, **57**, 1505–1509.

[38] U. Aschauer, Y. He, H. Cheng, S.-C. Li, U. Diebold and A. Selloni, *The Journal of Physical Chemistry C*, 2010, **114**, 1278–1284.

[39] G. Mattioli, F. Filippone, P. Alippi and A. Amore Bonapasta, *Phys. Rev. B*, 2008, **78**, 241201.

[40] E. Finazzi, C. Di Valentin, G. Pacchioni and A. Selloni, *The Journal of Chemical Physics*, 2008, **129**, 154113.

[41] B. Morgan and G. W. Watson, *Surface Science*, 2007, **601**, 5034–5041.

[42] S. Nosé, *Molecular physics*, 1984, **52**, 255–268.

[43] S. Nosé, *The Journal of chemical physics*, 1984, **81**, 511–519.

[44] W. G. Hoover, *Physical review A*, 1985, **31**, 1695.

[45] M. Horn, C. F. Schwerdtfeger and E. P. Meagher, *Zeitschrift fur Kristallographie*, 1972, **136**, 273–281.

[46] A. Vittadini, A. Selloni, F. P. Rotzinger and M. Grätzel, *The Journal of Physical Chemistry B*, 2000, **104**, 1300–1306.

[47] P. M. Oliver, G. W. Watson, E. Toby Kelsey and S. C. Parker, *J. Mater. Chem.*, 1997, **7**, 563–568.

[48] L. Agosta, F. Gala and G. Zollo, *AIP Conference Proceedings*, 2015, **1667**, 020006.

[49] L. Martínez, R. Andrade, E. G. Birgin and J. M. Martínez, *Journal of computational chemistry*, 2009, **30**, 2157–2164.

[50] K. Momma and F. Izumi, *Journal of applied crystallography*, 2011, **44**, 1272–1276.
